# Significance of zinc-solubilizing plant growth-promoting rhizobacterial strains in nutrient acquisition, enhancement of growth, yield, and oil content of canola (*Brassica napus* L.)

**DOI:** 10.3389/fmicb.2024.1446064

**Published:** 2024-09-27

**Authors:** Sabahet Jalal-Ud-Din, Nosheen Noor Elahi, Fathia Mubeen

**Affiliations:** ^1^Institute of Botany, Bahauddin Zakariya University, Multan, Pakistan; ^2^Soil and Environmental Biotechnology Division, National Institute for Biotechnology and Genetic Engineering (NIBGE-C, PIEAS), Faisalabad, Pakistan

**Keywords:** zinc-solubilizing rhizobacteria, canola, oil content, IAA synthesis, root colonization, biofilm production and sustainable agriculture

## Abstract

The present study was conducted with the aim to isolate, characterize, and identify the promising zinc-solubilizing rhizobacteria found naturally in the rhizosphere of canola (*Brassica napus* L.) plants. The study investigated the roles of these strains in nutrient acquisition and assimilation of extracellular molecules such as hormones and secondary metabolites. Ten isolated promising zinc-solubilizing strains (CLS1, CLS2, CLS3, CLS6, CLS8, CLS9, CLS11, CLS12, CLS13, and CLS15) were selected and characterized biochemically. Almost all the tested strains were Gram-positive, could fix nitrogen, and were positive for indole acetic acid, HCN, exopolysaccharides, and siderophore production. These effective zinc-solubilizing strains were identified through 16S rRNA gene sequencing. Based on the amount of solubilized zinc and halo zone diameter, four potent strains (CLS1, CLS2, CLS3, and CLS9) were selected for pot and field evaluation. Among all the identified bacterial genera isolated from the rhizosphere of the same host plant at different sampling sites, *Priestia aryabhattai* was found most abundant and found at all three sampling sites. The strains *Priestia megaterium*, *Staphylococcus succinus*, and *Bacillus cereus* were found at two different sites. *Bacillus subtilis* was found at only one site. These strains have a number of plant growth-stimulating characteristics as well as the ability to colonize plant roots successfully. The results indicated that inoculation of all these four zinc-solubilizing tested strains enhanced the plant growth, oil contents, and yield attributes of canola as compared to non-inoculated control with fertilizer levels. *Staphylococcus succinus* (CLS1) was first reported as a zinc solubilizer and associated with canola. *Priestia aryabhattai* (CLS2) and *Priestia megaterium* (CLS9) were found to be the best strains, with the most pronounced beneficial effect on canola growth and yield traits in both pot and field conditions. The site-specific dominance of these strains observed in this study may contribute toward decision-making for the development of specific inocula for canola. Therefore, identification of these strains could help in providing adequate amount of soluble zinc along with enhanced plant growth, yield, and oil content of canola.

## Graphical abstract

Isolation and identification of zinc-solubilizing rhizobacterial strains and their evaluation for pot and field trials.

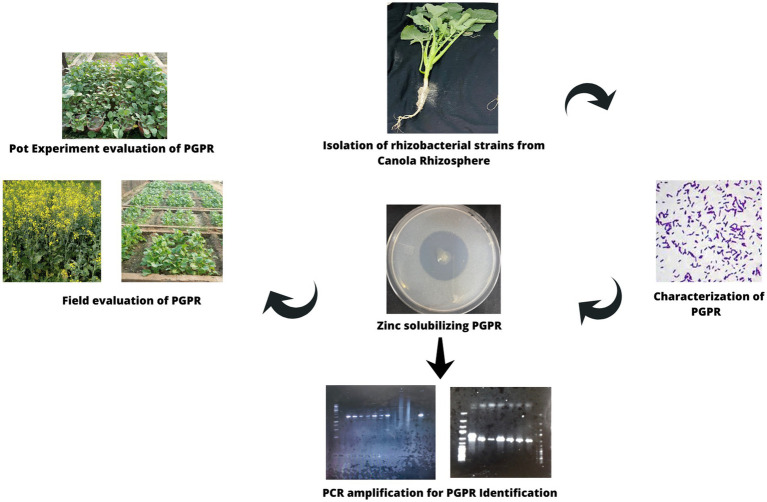

## 1 Introduction

Canola (*Brassica napus* L.) is an economic agricultural and major oilseed crop in Pakistan and South Asia with a rich source of nutritive qualities (Noreen et al., 2016). Since canola oil has a high percentage of unsaturated fatty acids, therefore it is a nutritious food source for both humans and animals (Nega and Woldes, 2018; Ahmad et al., 2020). Pakistan produced 0.46 million tons of edible oil domestically, which was not enough and fulfilled only 23% of the country’s requirement. Due to its increasing demand and production gap, Pakistan is facing a severe scarcity of edible oil. Soil productivity decreases as a result of rapid industrialization and vigorous cultivation practices. Due to losses caused by fixation, denitrification, leaching, precipitation, erosion, runoff, volatilization, and other processes, the majority of applied nutrients are not accessible to the plants (Gourley et al., 2012; Ghavami et al., 2016). Moreover, the improper utilization of synthetic fertilizers in agriculture is an important factor leading to changes in soil pH and ultimately has some adverse environmental impacts (Suthar, 2009; Reda and Hailu, 2017; Świątczak et al., 2023). The scientific community is seeking alternative methods that could substantially play a role in promoting and sustaining the production of oil seed crops. One promising method for reducing the adverse environmental impacts caused by improper use of chemical fertilizers is seed inoculation with plant growth-promoting rhizobacteria (PGPR). Rhizosphere-associated microorganisms may efficiently improve agricultural production quality and yield and significantly reduce the use of pesticides and chemical fertilizers (Premachandra et al., 2020). Zinc is one of the key micronutrients, and it is utilized by tissues in very small amounts (30–100 mg kg^−1^ dry matter of plant) for optimal plant development and reproduction, whereas Zn contents above 300 mg/kg are toxic for plants. It normally exists in Asian soil at a concentration of 69–89 mg kg^−1^ (Kiekens, 1995). Soil deficient in Zn contents has 10–30 mg kg^−1^ of zinc (Alloway, 2008). For plants, accessible forms of zinc occur in free ions (Zn^2+^ and ZnOH^+^) and organically complexed Zn solution in soil. Zinc precipitates as sulfides and forms complexes with organic matter in organic soil due to its achalcophile components (Vodyanitskii, 2010).

Zinc deficiency is a well-known problem that affects both plants and humans (Yadav et al., 2023). Its shortage in plants retards nitrogen metabolism and photosynthesis, causes a reduction in fruit development and flowering, limits the synthesis of phytohormones and carbohydrates, and delays crop maturity, leading to decrease in nutritional quality of grains and crop yield (Singh et al., 2005). Additionally, zinc activates many enzymes and plays a key role as cofactor in the enzyme tryptophan synthetase, which is involved in the production of IAA (Hafeez et al., 2013). Plants can absorb zinc in the form of the divalent cation Zn^2+^ and chelate form, although most of the zinc in the soil is found in insoluble forms Zn(OH)_2_, ZnO, and ZnCO_3_ (Abaid-Ullah et al., 2015). The cultivation of zinc-solubilizing bacteria is a significant substitute for chemical fertilizers, which stabilize soil characteristics over time by gradually releasing the nutrients found in the soil and preserving the soil microflora (Dinesh et al., 2018). Through bioaugmentation of microbial isolates with a capability to solubilize insoluble Zn compounds, the unavailable Zn compounds can be transformed back into accessible form (Saravanan et al., 2007; Yadav et al., 2022). Instead of inadequate Zn availability in soil, low Zn solubility is the primary cause of the widespread Zn deficit in crops (Iqbal et al., 2010; Barbagelata and Mallarino, 2013; Gontia-Mishra et al., 2016). A number of PGPR have been reported for their ability to solubilize the insoluble form of zinc in soil (Hussain et al., 2015; Rahman et al., 2024). Inoculating crops with Zn-solubilizing isolates may not only help us to resolve the issue of malnutrition through enhancing the nutritional contents in grains, but they may also serve as essential equivalents for zinc fertilizers. Zinc PGPR regulates the soil characteristics for a longer period of time by gradually releasing the nutrients found in soil with inaccessible Zn concentrations (Sindhu et al., 2019). Hence, exploitation of zinc-solubilizing bacteria for zinc fortification in food grains like canola as well as for alleviating the zinc deficiency in canola plants could be a promising agronomical approach. The plant’s growth is facilitated by rhizobacteria directly by fixing atmospheric nitrogen, simulating the synthesis of plant hormones, and alleviating nutrient uptake through zinc solubilization (Akbar et al., 2019; Kumar et al., 2019). They also facilitate the plant growth indirectly by preventing the deleterious effects of phytopathogenic organisms through antibiotics, hydrolytic enzymes (amylases, cellulases, proteases, and dehydrogenases), volatile compounds (hydrogen cyanide and ammonia), and siderophores (Kumar and Dubey, 2012; Backer et al., 2018; Gouda et al., 2018). Indole acetic acid has a direct effect on plant cell division, differentiation, and extension (Shoebitz et al., 2009). It promotes root development, xylem formation, and seed germination. Moreover, it regulates photosynthesis, formation of different metabolites, and pigment production. However, the accumulation of IAA that has been released by soil bacteria could influence the naturally occurring IAA reservoir of plants (Afzal et al., 2015). In agro-biotechnology industry, *Bacillus* is an adaptable genus and one of the most commercially exploited bacteria. Members of the genus *Bacillus* are considered to have a number of beneficial characteristics that support plants through protection from pathogens, recovery of nutrients by cycling of nutrients, and overall enhancement in growth by phytohormone production (Mumtaz et al., 2020). These strains could be employed as biofertilizers to enhance growth and productivity of agricultural crops, giving a replacement to the application of synthetic nitrogen fertilizers (Defez et al., 2017). These microorganisms promote plant growth and maximize yield of crops (Babalola, 2010; Kumar et al., 2016; Kumar et al., 2017). Microbes have evolved a number of iron acquisition mechanisms for their existence and adaptation to environment, helping the plant overcome this challenge by obtaining iron. One of these mechanisms is the formation of these molecules known as siderophores, small organic compounds synthesized by bacteria in iron-limiting conditions to increase the iron sequestration ability (Dimkpa et al., 2009; Aznar and Dellagi, 2015; Li et al., 2016). Most extensively studied genera *Bacillus* have multiple growth-promoting characteristics, such as the ability to solubilize zinc, fix nitrogen, produce antibiotics, siderophores, and secondary metabolites that inhibit soil-borne plant pathogens (Chen et al., 2007; Mumtaz et al., 2018; Shahid et al., 2022). Extracellular polymeric substances (EPS) are polymers of carbohydrate released by a wide range of zinc-solubilizing rhizobacteria. These plant-associated rhizobacteria are additionally employed to promote systemic tolerance by the synthesis of EPS (Naseem et al., 2022). Surface-associated microbial cells that form biofilms are enveloped in a self-produced EPS that mostly contains polysaccharide, extracellular DNA, lipids, and proteins (Flemming and Wingender, 2010). Biofilm PGPR showed exceptionally high levels of IAA generation, nitrogenase activity, phosphate solubilization, and siderophore formation (Zhang et al., 2015). The prospect of zinc-solubilizing rhizobacteria in agriculture is gradually increasing since it presents an appealing substitute to the excessive use of zinc fertilizers, pesticides, and other supplements. A great deal of research work has been done to isolate and characterize rhizobacteria that solubilize zinc, and numbers of strains are identified for other crops. However, canola has garnered little attention in this context even though it is the world’s second largest oilseed crop after soybeans (Martínez-Hidalgo et al., 2021). Previously, limited work has been done on isolation and identification of microbial flora of canola (Valetti et al., 2018). To date, most research works have focused on Gram-negative endophytic bacteria in case of canola (Farina et al., 2012). Scant information has been found in regards to Gram-positive endophytes impacting canola growth (Jimenez-Gomez et al., 2020). Hence, the importance of indigenous bacterial isolates in increasing canola crop productivity prompted us to investigate the ability of locally isolated zinc-solubilizing PGPR, which would perform better due to being adaptable to local climatic conditions. Therefore, it is essential to explore and discover the region-specific plant growth-promoting rhizobacterial strains because they will be more effective, best fitting and unique for promoting crop growth and yield in that region. Keeping in view the significance of Gram-positive rhizobacteria in enhancing growth and yields of canola, the current research was conducted to isolate the indigenous bacteria from the rhizosphere of canola plants that are host-specific and identify the novel strains through 16S RNA gene amplification and sequencing. Moreover, we also evaluated the selected PGPR for plant growth-promoting (PGP) attributes such as zinc solubilization, root colonization, siderophore, biofilm, EPS, and phytohormone (IAA) production. This research is also focused on exploiting these promising zinc-solubilizing strains for improving growth and yield attributes such as seed oil level and fattyacid profile of canola for enhanced canola production under pot and field conditions.

## 2 Materials and methods

The present study was carried out in the Microbial Physiology and Biocontrol Lab of Plant Microbiomes, Soil and Environmental Biotechnology Division of NIBGE, Faisalabad, Pakistan.

### 2.1 Bacterial isolation

Canola (*Brassica napus* L.) plants along with rhizosphere were collected from canola fields in the different districts of Punjab Province, Liyyah (30°46′02.0″N 70°55′16.2″E), Dera Ghazi Khan (30°09′26.8″N,70°43′48.3″E) and Faisalabad (31°240 32.2100″N 73° 040 5.8600″E), Pakistan. Rhizosphere soil suspensions were diluted to a concentration of 10^−1^ to get single colonies. Fifty μl of these dilutions were then spread onto Luria-Bertani (LB) agar medium (tryptone 10 g, NaCl 5 g, yeast extract 5 g, nutrient agar 20 g, distilled water 1 L, pH 7) plates and incubated at 28 ± 2°C for 48 h. From the liquid cultures, 0.8 ml of bacterial cultures that had been incubated for 48 h were combined with 0.2 ml of sterile glycerol and then preserved at −80°C for further investigation.

### 2.2 Morphological cultural characterization

Bacterial isolates were characterized on the basis of their size, shape, color, margin, pigmentation, and surface. These characteristics were documented following the guidelines provided by Holt et al. (1994) according to Bergey’s Manual of Determinative Bacteriology. The standard protocol outlined by Dubey and Maheshwari (2011) was used to perform Gram staining for the unknown bacterial isolates.

### 2.3 Production of Indole-3-acetic acid by bacterial isolates

Bacterial isolates were evaluated for their capability to synthesize indole-3-acetic acid (IAA) employing a colorimetric detection assay in liquid culture by utilizing the Salkowski reagent as exemplified by Shao et al. (2015). Each strain cultures were incubated in 5 ml of LB broth medium at 28°C for 48 h, after which 20 μl of culture cells were placed in Dworkin and Foster’s (1958) salt minimal media enriched with and without L-tryptophan (5 mg L^−1^) at 28°C for 48 h on an orbital shaker (Thermo Scientific^™^, MaxQ^™^, 40,000^®^). By measuring the absorbance at 535 nm, spectrophotometer (GBC-Avanta AA series) was used to quantify the intensity of the pink-red color. The standard curve was constructed through IAA production (μg ml^−1^) by bacterial isolates and standard solution. Three replicates of each bacterial strain were employed, and the experiment was repeated.

### 2.4 Nitrogenase activity

Nitrogen fixation was evaluated to test the efficiency of bacterial strains to convert nitrogen gas from the atmosphere into nitrate that is accessible by plants. Nitrogenase activity was determined by employing nitrogen-free medium (Hafeez and Malik, 2000) with three replicates. Quantitative estimation of nitrogen-fixing capacity (nmol h^−1^ mg^−1^) of 10 rhizobacterial isolates was tested employing the acetylene reduction assay (ARA), according to the protocol provided by Sajjad Mirza et al. (2001).

### 2.5 Iron Solubilization assays

The chrome azurol sulfonate (CAS) assay is a valuable method for evaluating prospective biocontrol microorganisms that produce siderophores. The tested isolates were subjected to CAS assay by following the protocol as reported by Schwyn and Neilands (1987). The CAS assay is based on the color change around the microbial colony of CAS iron complex from blue to orange following the association of the bound iron by siderophores. The magnitude of the yellowish-orange haloes around rhizobacterial colonies was suggested to be positive for siderophore production.

### 2.6 Exopolysaccharide synthesis

Tested isolates were evaluated for exopolysaccharide (EPS) production by Gerhardt (1994) on yeast extract mannitol agar medium (mannitol 10 g, K_2_HPO_4_ 0.5 g, MgSO_4_·7H_2_O 0.2 g, NaCl 0.1 g, yeast extract 0.5 g, agar 15 g, distilled water 1 L). For 3 days, plates were incubated at 28 ± 2°C. The production of mucoid was considered as positive for EPS.

### 2.7 Hydrogen cyanide production

All of the isolates have been analyzed for the formation of HCN employing the Castric (1975) methodology. Bacterial strains were spread on nutritional media plates containing 4.4 g/L glycine. On the lid of the Petri plate, a piece of sterilized filter paper soaked in 1% picric acid and 2% sodium carbonate solution was inserted. These plates were incubated for 4 days at 28–30°C. The transformation of the color from orange to reddish-brown suggested that bacterial strains were producing HCN.

### 2.8 Evaluating the rhizobacterial isolates for hydrolytic enzyme synthesis

In this study, bacterial strains were analyzed for their ability to produce hydrolytic enzymes such as protease, cellulase, and amylase.

#### 2.8.1 Protease

Bacterial isolates were evaluated for their ability to produce proteolytic enzymes on the skim milk agar (3% v/v) media by following the standard procedures of Chang et al. (2009).

#### 2.8.2 Cellulase

Cellulose degradation ability of bacterial isolates was detected by streaking culture on cellulose Congo red agar medium (MgSO_4_·7H_2_O 0.25 g, K_2_HPO_4_ 0.5 g, cellulose 2 g, Congo red 0.2 g, gelatin 2 g, agar 15 g, distilled water 1 L, pH 6.8–7.2). Discoloration of Congo red indicator in an agar medium by cellulolytic bacteria provides rapid and sensitive screening (Gupta et al., 2012).

#### 2.8.3 Amylase

The hydrolysis of starch by bacterial strains was examined using starch agar medium (starch 20 g, tryptone 10 g, NaCl 5 g, yeast extract 5 g, 20 g nutrient agar, distilled water 1 L, pH 7) and incubated for 48–72 h at 28 ± 2°C. Following incubation, starch agar plates were flooded with autoclaved Lugol’s solution [iodine (5%), KI (10%), distilled water (100 ml)], allowed to keep for 1 min, and then drained off. After the reaction of iodine with starch, a blue product is formed. This blue color abruptly vanishes. Following that, colorless area surrounding the bacterial colonies showed starch hydrolysis (Gupta et al., 2003).

### 2.9 Calcium solubilization assay

The calcium-dissolving bacteria (CDB) differentiating media (NaCl 5 g, CaCO_3_ 5 g, glucose 5 g, K_2_HPO_4_ 0.4 g, MgSO_4_ 0.01 g, peptone 1 g, (NH_4_)_2_SO_4_ 0.05 g, yeast extract 1 g, agar 7.5 g, distilled water 1 L, pH 7.0) supplemented with calcium in the form of calcium carbonate (Peper et al., 2022). It was used to identify the bacterial isolates having ability to solubilize insoluble calcium and convert it into plant-available soluble form. The freshly grown bacterial culture was inoculated on the CDB medium plates and incubated for 3–4 days at 28 ± 2°C. Clear zones were observed around the bacterial colony, indicating that the bacterial strains solubilize calcium complexes.

### 2.10 Zinc solubilization assay

Ten strains were examined in Mineral Salt Medium (MSM) described by Saravanan et al. (2008) to determine the zinc solubilization capability. Insoluble zinc ZnO was added to the medium (NaCl 1 g, CaCl_2_ 0.1 g, KH_2_PO_4_ 1 g, K_2_HPO_4_ 0.1 g, MgSO_4_ 0.5 g, ZnO 0.1%, yeast extract 4 g, agar 18 g, distilled water 1 L, pH 7.0) and autoclaved at 121°C for 30 min. Overnight-produced fresh bacterial cultures were spot inoculated in triplicate on the sterilized media containing Petri plates with sterile toothpicks. The spotted plates were incubated in the dark for 1 week at 28 ± 2°C to detect the formation of distinct halo zones surrounding colonies. After 7 days, the diameter of the halo zone around the colonies and the diameter of the colonies were measured. The zinc solubilization efficiency (ZSE) and zinc-solubilizing index (ZSI) were determined using the methods of Khanghahi et al. (2018) and Rafique et al. (2022). Zinc solubilizers having the highest value of ZSE are believed to be efficient.

$$ZSI=\frac{\text{Colony Diameter}+\text{Halo Zone Diameter}\;}{\text{Colony Diameter}}$$

$$ZSE=\frac{\text{Diameter of halo zone of solubilization}}{\text{Colony Diameter}}\times 100$$

Quantitative zinc solubilization by rhizobacterial isolates was estimated by using a mineral salt medium broth provided with 0.1% insoluble zinc compounds. Overnight grown culture of zinc-solubilizing isolates (1 × 10^4^ CFU ml^−1^) was inoculated into each flask containing 50 ml of MSM broth and then incubated for 2 weeks at 30°C, then on rotary shaker for 48 h at 180 rpm. After 14 days of incubation, 10 ml of samples were removed and centrifuged for 10 min at 10,000 rpm and filtered using Whatman filter paper of 11 μm (Cytiva, Whatman1001-045 US). Following that, 2 ml of supernatant was transferred to a 100-ml flask, and distilled water was used to dilute the volume up to 100 ml. The culture supernatant was fed directly to atomic absorption spectrophotometer at 230 nm to quantify the level of soluble zinc (Dinesh et al., 2018). Highly zinc-solubilizing strains were picked for further evaluation. A medium with no culture was used as a control. The amount of solubilized zinc was estimated as (mgL^−1^ = ppm) culture by subtracting the soluble Zn of the inoculated sample from the equivalent non-inoculated control. A pH meter was also used to measure the pH of the culture and control. The experiments were conducted in triplicate.

### 2.11 Evaluation of rhizobacterial strains for potassium solubilization

Initially, selected bacterial strains were subjected to K solubilization screening test based on halo zone production on modified Aleksandrov’s agar medium via spot test method following the modified standard methodology of Meena et al. (2015). Spot-inoculating Petri plates were incubated for 7 days at 28 ± 2°C. The halo zone appearance around the bacterial colonies indicated the positive results.

### 2.12 Phosphate solubilization assays

To detect the qualitative phosphate solubilization potential of isolated bacterial strain, liquid Pikovskaya’s agar medium [yeast extract 0.5 g, dextrose 10 g, Ca(PO_4_)_2_ 3 g, (NH_4_)_2_SO_4_ 0.5 g, KCl 0.2 g, MgSO_4_ 7H_2_O 0.1 g, MnSO_4_ 0.0001 g, Fe-EDTA 0.0001 g, CaCo_3_ 0.3 g, agar 18 g, distilled water 1 L, pH 7] was used in triplicate (Pikovskaya, 1948). After sterilization and pouring, bacterial strains were spot inoculated on the Pikovaskaya’s agar medium and incubated at 28 ± 2°C for 5–7 days. Halos zone formed around the bacterial colony after the incubation. This halo zone formation indicated the positive results.

### 2.13 PCR amplification of 16S rRNA gene sequencing and phylogenetic analysis

The selected promising rhizobacterial isolates having growth endorsing characters in canola were identified through amplification, sequencing, and bioinformatics analysis of its 16S rRNA gene sequence. For this purpose, crude DNA of the selected isolates was extracted from the bacterial isolates by CTAB method (Wilson, 1987). DNA concentration was determined through the comparison of the isolated DNA with the DNA intensity marker on agarose gel (0.8%), and the DNA was preserved at −20°C. For amplification, the 16S rRNA gene with PCR reagents was amplified in a thermalcycler (SuperCycler, Model: SC300G R2, Australia) using the 16S primers: fD1-5′-AGA GTT TGA TCC TGG CTC AG-3′ and rD1-5′-AAG GAG GTG ATC CAG CC-3′. The thermocycling conditions involved an initial denaturation at 94°C for 3 min, followed by 30 cycles of 94°C for 30 s, 53°C for 30 s, and 72°C for 1 min and a final extension at 72°C for 5 min. Amplified PCR product (1.5 kb) was eluted from the gel and cleaned using commercial QIA Quick Gel Extraction Kit (QIAGEN Sciences, Maryland 20,874, USA). Amplification product (1,500 bp) was sent to Macrogen Laboratories Inc., Seoul, Korea^1^ for sequencing. Basic Local Alignment Search Tool (BLAST) was used to identify nucleotide-related sequence similarities which were obtained from the GenBank database “National Centre for Biotechnology Information (NCBI) database https://blast.ncbi.nlm.nih.gov/Blast.cgi.” Species were assigned based on the highest sequence identity, highest E-value, and coverage. Phylogenetic relationship between the representative bacterial species and identified strains based on 16S rRNA gene sequences was developed with the ClustalW program as described by Roohi et al. (2012). A phylogenetic tree was constructed by maximum likelihood method among different isolates using MEGA X version 11.0.13 (Kumar et al., 2018). Confidence in the tree topology was evaluated by bootstrap analysis with 1,000 replicates (Tamura et al., 2013). The isolates identified in the current study are indicated in bold text. The comparison of almost complete gene sequences has been used to establish taxonomic relationships between prokaryotic strains with 98.65% similarity currently recognized as the cutoff for delineating species (Kim and Chun, 2014).

### 2.14 Biofilm formation

Zinc-solubilizing rhizobacterial strains were evaluated for their ability to form biofilm using the procedure described by Haque et al. (2020). Overnight-grown cultures in LB broth having optical density (OD_660_) reached 0.7–0.8. Then 1 ml of culture of each strain was centrifuged at 10,000 rpm for 10 min. The pellet was diluted (ca. 106 CFU/ml). Glass test tubes were filled with 5-ml salt-optimized broth plus glycerol (SOBG) medium (tryptone 20 g, KCl 0.186 g, MgSO_4_.7H_2_O 2.4 g, NaCl 0.5 g, yeast extract 5 g, 40% glycerol 50 ml, H_2_O 1,000 ml, pH 7.00) and 50 μl of each bacterial cultures were then kept in an incubator at 28°C. Air Liquid (AL) biofilm-producing rhizobacteria were selected after 72 h. Biofilm of selected strains was removed from glass test tubes and washed with sterile water three times. After that, 1 ml of sterile distilled water and 14 glass beads (3 mm) were added to each glass tube. By vortexing at high speed for 50 s, biofilm was detached. Then optical density was measured by reading the absorbance at 660 nm with spectrophotometer (CamSpec M350 double beam UV–visible). Biomass of biofilms was estimated by employing the methodology presented by Mosharaf et al. (2018). Three replicates for every rhizobacterial isolate were used, and the experiment was repeated.

### 2.15 Root colonization assay

Roots of canola plants that were growing in sterilized sand were used in this assay. Surface-sterilized canola roots (200 mg) were macerated and ground in 6 ml sterilized distilled water with sanitized mortar and pestle. Subsequently, tubes were shaken at 250 rpm for 20 min at room temperature. After shaking, suspensions were serially diluted 10^−1^ to 10^−7^. A volume of 20 μl of diluted root suspension was spot inoculated into sterilized LB media Petri plates and incubated at 28 ± 2°C for 48 h. After that, the bacterial population was counted in terms of colony-forming units (CFU). The number of bacteria colonizing the root was calculated as CFU/mg root. Logarithm of CFU/mg of root was taken.

$$\text{logCFU}=\frac{\text{No}.\;\text{of colonies}\times \text{Total dilution factor}}{\;\text{mg}\;\text{of root}}$$

### 2.16 Pathogenicity test

The pathogenicity test was performed employing the methodology described by Chahad et al. (2012). The bacterial isolates were spot inoculated on blood agar plates supplemented with 5% (v/v) sheep blood to determine their hemolytic activity (peptone 10 g, NaCl 5 g, beef extract 3 g, agar 15 g, distilled water 1 L). Sheep blood was added after autoclaving and before pouring it onto the plates. Plates were incubated at 28 ± 2°C for 48 h. The findings were recorded for the appearance of zones with difference in color.

### 2.17 PGP potential of zinc-solubilizing strains in pot trial

The zinc-solubilizing ability of these selected rhizobacterial strains was checked in a pot experiment. During the canola growing season (October–March), pot trial was carried out in a net house under natural temperature and light conditions using tap water at NIBGE, Faisalabad. The experiment was conducted in sterilized sand with three replicates. Autoclave sand (10 kg) was filled in 18 cm diameter pots. Six treatments were planned for the experiment, including inoculation of four most promising zinc-solubilizing strains, CLS1, CLS2, CLS3, and CLS9, and two non-inoculated control (with or without fertilizer treatment) with three replicates per treatment. A single colony of each strain was added to four sterilized conical flasks holding 250 ml of nutrient broth and incubated for 7 days at 28°C in a rotary shaking incubator. Surface sterilization of canola seeds of two cultivars was performed by first immersing them in ethanol (95%) for 1 min, followed by dipping in 0.2% solution of HgCl_2_ for 3 min. Then the seeds of canola were washed three times with sterile distilled water. Sterilized filter mud was used as carrier material. For one acre of seedlings, 1 kg of carrier material was used. After sterilizing the filter mud, it was placed in sterile sealed plastic bags. The fresh culture broth (250 ml) of selected rhizobacterial strains (1 × 10^4^ CFU ml^−1^) was injected into the bag of sterile mud. After that, canola seeds were immersed in these bags and then left for 2 h before sowing in pots. In the net house, pots were arranged in a complete randomized design. Eight coated seeds were sown at equal intervals in every pot and watered regularly. Each treatment was used in triplicate. After 10 days of germination, plants were thinned to a maximum of 5 plants in every pot.

### 2.18 Field evaluation of zinc-solubilizing strains for their PGP potential

Two field experiments were carried out in Dera Ghazi Khan to investigate the efficiency of zinc-solubilizing strains in increasing canola plant growth and crop production under field conditions. Experiments were planned in a randomized complete block design (RCBD), including three replicates. This trial has same non-inoculated and inoculated treatments and replicates that were evaluated in the pot experiment. Three plants from each treatment were selected at random to determine the impact of the treatments on crop growth. To determine the dry weight of shoots and roots, samples from each treatment and replication were dried in an oven (FELISA, model 242-A^®^) at 67°C. The number of grains/pod, number of pods/plant, grain yield/plant, and weight of thousand seeds were all collected and statistically examined. All data were acquired using standard methods (Namvar and Khandan, 2014).

### 2.19 Oil quality analysis

The protein, total seed oil contents, and fatty acid profile were tested by the method illustrated by da Silva Medeiros et al. (2022). Protein and oil content of seeds were determined using oilseeds calibrated near-infrared reflectance (NIR) spectroscopy (Model: Perten DA 7250, USA) at Hi-Technology Laboratory of Oilseeds Research, Ayub Agricultural Research Institute, Faisalabad, Pakistan.

### 2.20 Statistical analysis of data

For statistical analyses, the data regarding *in vitro* experiment were compared through one-way analysis of variance (ANOVA). The data regarding *in vivo* trials were statistically analyzed, and the Statistix 8.1 computer software was employed to evaluate the significance among treatments. The obtained means of three replications were subjected to the Tukey test at 5% probability (Steel et al., 1997).

## 3 Results

### 3.1 Isolation, purification, and screening of PGPR

The present study was conducted to investigate an eco-friendly method of using plant growth-promoting rhizobacteria as a bioinoculant to enhance canola nutrition, growth, and yield. The main objective of this study was to isolate and characterize Zn-solubilizing strains from different cities in Punjab, Pakistan. A total of 84 rhizobacterial strains were isolated from the canola rhizosphere. These strains were purified on separate fresh agar plates and stored at 4°C for further experimentations.

### 3.2 Morphological and biochemical characterization of selected rhizobacterial strains for PGP attributes

Out of 84, only 10 indigenous potent zinc-solubilizing rhizobacterial isolates were selected and subjected to detailed characterization for their plant growth-enhancing attributes. Morphologically, most of these rhizobacterial isolates were motile, Gram-positive, and rod-shaped (Table 1). To determine the plant growth-stimulating characteristics of the most auspicious rhizobacterial strain, biochemical characterization was carried out, and potent isolates were selected for screening trials. These rhizobacterial strains were evaluated for zinc, phosphate, potassium, and calcium solubilization, HCN, siderophore, exopolysaccharide, protease, and cellulose production, and starch hydrolysis, as depicted in Table 2. The potential of rhizobacterial isolates to solubilize phosphate in the Pikovskaya medium was examined. Seven rhizobacterial isolates that showed phosphate solubilization are listed in Table 2. Among the 10 rhizobacterial isolates, CLS8 and CLS11 were not able to produce siderophore, while the other eight isolates showed positive results in producing siderophores. Four rhizobacterial isolates, CLS8, CLS9, CLS11, and CLS12, could not produce HCN; however, all other tested isolates were well capable of producing HCN (Table 2). Amylase production of isolates was determined by starch hydrolysis test. Production of exopolysaccharides was checked by the formation of precipitation in broth inoculated with the selected isolates. Some of the isolates CLS2, CLS6, CLS8, and CLS11 produced exopolysaccharides and showed positive results, while the rest of the isolates were unable to produce exopolysaccharides (Table 2).

**Table 1:** Morphological and cultural characteristics of rhizobacterial strains isolated from Canola (*Brassica napus* L.) rhizosphere.

Sr. No.	No. of isolates	Colony size	Colony colour	Colony margin	Colony shape	Surface	Motility	Gram reaction	Shape	Pigmentation
1.	CLS1	Small	White	Entire	Round	Smooth, opaque	Nonmotile	Positive	cocci	White
2.	CLS2	Large	Off white	Entire	Round	Smooth, opaque	Motile	Positive	Rod	Non pigmented
3.	CLS3	Medium	milky white	Irregular	Round	Rough, opaque	Nonmotile	Positive	Rod	Fuzzy White
4.	CLS6	Large	Off white	Entire	Round	Smooth, opaque	Motile	Positive	Rod	Non pigmented
5.	CLS8	Large	Grey	Irregular	Round	Rough, opaque	Motile	Positive	Rod	Dull Grey
6.	CLS9	Medium	White	Regular	Irregular	Smooth, opaque	Motile	Positive	Rod	Cream
7.	CLS11	Large	Grey	Irregular	Round	Rough, opaque	Motile	Positive	Rod	Dull Grey
8.	CLS12	Medium	Dull white	Regular	Irregular	Smooth, shiny	Motile	Positive	Rod	Cream
9.	CLS13	Large	Off white	Entire	Round	Smooth, opaque	Motile	Positive	Rod	Non pigmented
10.	CLS15	Small	White	Entire	Round	Smooth, opaque	Nonmotile	Positive	cocci	White

**Table 2:** Biochemical and biocontrol activities of PGPR isolates from Canola (*Brassica napus* L.)

Isolates	Zinc solubilization	Phosphate solubilization	Potassium solubilization	Calcium solubilization	HCN production	Siderophore production	EPS production	Protease production	Cellulose production	Starch hydrolysis
CLS1	+	−	−	+	+	+	−	+	+	+
CLS2	+	−	−	−	+	+	+	+	+	+
CLS3	+	−	−	−	+	+	−	+	+	+
CLS6	+	−	−	−	+	+	+	+	+	+
CLS8	+	+	−	−	−	−	+	+	+	+
CLS9	+	+	−	−	−	+	−	+	+	+
CLS11	+	+	−	−	−	−	+	+	+	+
CLS12	+	+	−	−	−	+	−	+	+	+
CLS13	+	−	−	−	+	+	−	+	+	+
CLS15	+	−	−	+	+	+	−	+	+	+

Zinc solubilizing rhizobacterial isolates having multiple plant growth promoting characteristics; All *in vitro* tests were repeated twice for verification of results with three replicates each time; The symbol, + represents the presence of the traits while the symbol, −shows the absence of the trait.

### 3.3 Zinc solubilization by rhizobacterial isolates from canola

**Figure 1:**
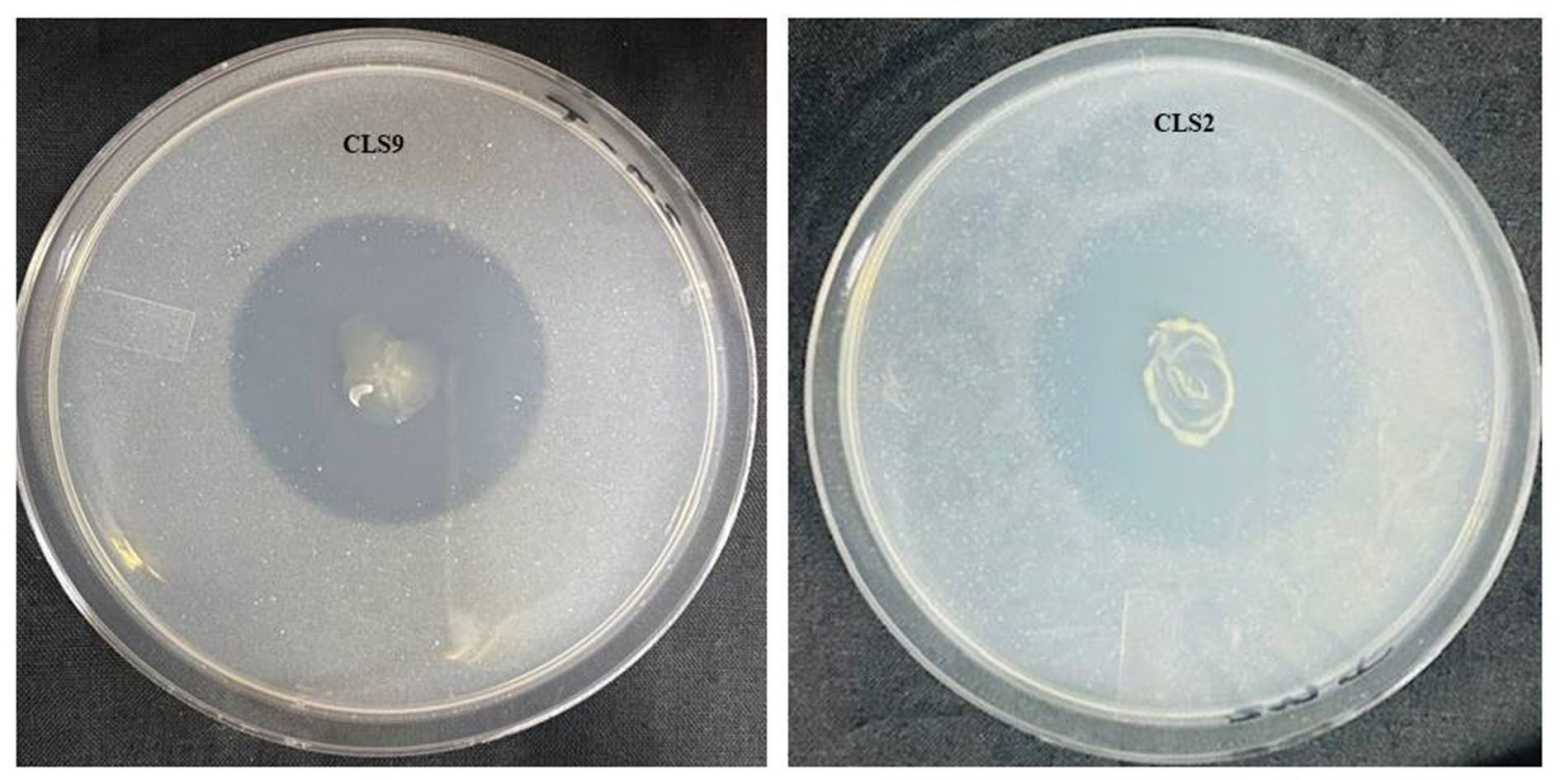
Solubilization of ZnO during *in vitro* assay by selected potent zinc-solubilizing strains after 5 days of incubation. The large clear halo zones around the colonies of *Priestia aryabhattai* (CLS2) and *Priestia megaterium* (CLS9) indicate the ZnO solubilization.

In the current study, 10 isolates (CLS 1, CLS 2, CLS 3, CLS 6, CLS 8, CLS 9, CLS11, CLS12, CLS 13, and CLS15) demonstrated high solubilization of insoluble Zn compound (ZnO) during agar plate assay. Among 10 rhizobacterial isolates, CLS2 from the *Bacillus* genus showed the greatest ability for Zn solubilization. *In vitro* analysis of selected isolates for their ability to solubilize ZnO indicated that zinc solubility ranged from 7.90 to 29.63 mm halo zone diameter (Table 3). Data regarding solubilization efficiency (ZSE) and zinc solubilization index (ZSI) based on halo zone and colony diameter indicated that the most effective isolates were CLS2, CLS6, and CLS13, which were statistically non-significant to each other (Table 3). The isolate CLS12 also showed good Zn solubilization zone and was followed by CLS9. The inoculation of isolates CLS8 and CLS11 produced lowest solubilization zone. Rhizobacterial isolates that displayed an increase in activity of ZnO solubilization in solid media were subjected to inoculation in minimal salt medium broth modified with ZnO to evaluate their effect on pH. Data regarding pH decrease (Table 3) showed that a large number of isolates decreased the pH of broth, but maximum drop in pH of medium up to 4.63 ± 0.02 was noted with inoculation of CLS2, followed by CLS9, CLS6, and CLS13. While isolate CLS11 displayed lowest decline in pH (Table 3). After the identification of these strains, four different strains, CLS1, CLS2, CLS3, and CLS9, were selected as highly efficient Zn solubilizers and recommended for further pot and field studies (Figure 1).

**Table 3:** Zinc solubilization by rhizobacterial strains isolated from Canola (*Brassica napus* L.) rhizosphere.

Isolates	Bacterial colony diameter (mm)	Zinc halo zone diameter (mm)	Zinc solubilization efficiency (ZSE)	Zinc solubilization index (ZSI)	pH of isolates
CLS1	5.40 ± 0.26 ^d^	12.00 ± 0.47 ^d^	222.43 ± 2.20 ^d^	3.21 ± 0.02 ^d^	5.17 ± 0.05 ^b^
CLS2	8.60 ± 0.05 ^a^	29.63 ± 0.37 ^a^	344.55 ± 2.32 ^a^	4.44 ± 0.02 ^a^	4.63 ± 0.02 ^c^
CLS3	6.13 ± 0.08 ^c^	12.43 ± 0.35 ^d^	202.63 ± 2.81 ^e^	3.02 ± 0.02 ^e^	5.05 ± 0.04 ^b^
CLS6	8.70 ± 0.1 ^a^	29.80 ± 0.23 ^a^	342.56 ± 2.10 ^a^	4.42 ± 0.02 ^a^	4.68 ± 0.03 ^c^
CLS8	6.93 ± 0.14 ^b^	7.90 ± 0.07^e^	113.99 ± 1.33 ^f^	2.13 ± 0.01 ^f^	5.70 ± 0.04 ^a^
CLS9	8.33 ± 0.14 ^a^	23.13 ± 0.14 ^c^	277.70 ± 3.08 ^c^	3.77 ± 0.02 ^c^	4.65 ± 0.04 ^c^
CLS11	6.90 ± 0.05 ^b^	7.93 ± 0.11 ^e^	114.62 ± 0.71 ^f^	2.14 ± 0.005 ^f^	5.75 ± 0.03 ^a^
CLS12	8.16 ± 0.12 ^a^	25.10 ± 0.17 ^b^	307.42 ± 2.51 ^b^	4.07 ± 0.02 ^b^	4.78 ± 0.04 ^c^
CLS13	8.53 ± 0.12 ^a^	28.80 ± 0.20 ^a^	337.57 ± 2.90 ^a^	4.37 ± 0.02 ^a^	4.71 ± 0.02 ^c^
CLS15	5.46 ± 0.17 ^cd^	12.36 ± 0.29 ^d^	226.36 ± 3.13 ^d^	3.25 ± 0.03 ^d^	5.14 ± 0.05 ^b^
CVC	0.70	1.36	12.16	0.11	0.20

Rhizobacterial Isolates were incubated for zinc solubilization on agar media amended with ZnO for 7 days at 28 ± 2°C, Zinc halo zone diameter, ZSE and ZSI were determined using diameters of zinc halo zone and bacterial colony diameter; whereas, solubilized zinc concentration by isolates and pH drop in broth was recorded after10 days of incubation; Data presented are the mean of three replicates ± standard error and Tukey HSD test was performed at 5% (*p* ≤ 0.05) probability level; CVC, critical value for comparison.

### 3.4 Screening of zinc-solubilizing rhizobacterial isolates

Our results for evaluating selected isolates for their potential to solubilize Zn in plate assay demonstrated that all screened rhizobacterial isolates were also positive for Zn solubilization in liquid media. Promising rhizobacterial isolates of zinc solubilization in solid media were inoculated in broth with ZnO for quantitative Zn solubilization assessment. Soluble zinc content values were noted from atomic absorption spectrophotometer. Results obtained revealed that these isolates, CLS2, CLS6, and CLS13, were statistically similar to each other, followed by CLS9 and CLS12, after 7 days of incubation (Table 4). Minimum quantity of solubilized zinc was acquired with the inoculation of isolates CLS8 and CLS11, respectively, which were non-significant with each other. The IAA generation assay revealed that eight evaluated rhizobacterial strains exhibited the ability of IAA production, while two strains, CLS8 and CLS11, did not show IAA production. In fact, with L-tryptophan, the highest production of IAA was synthesized by CLS6, followed by strains CLS2, CLS13, and CLS12, and statistically non-significant with each other. Strain CLS9 also showed remarkable IAA production without L-tryptophan. Similarly, strains CLS1 and CLS15 were non-significant with each other (Table 4). After NFM media test, all the rhizobacterial isolates were subjected to nitrogenase activity in acetylene reduction assay (ARA). Nitrogen-fixing ability in these rhizobacterial isolates was further validated by gas chromatography. The ARA results indicated that out of 10, eight bacterial isolates were excellent for nitrogenase ability. The highest value of nitrogen fixation was noticed from strains CLS2 and CLS6, which were statistically similar and non-significant with each other (Table 4). *In vitro* root colonization tests indicated that some isolates are more potent root colonizers than others. The bacterial cell counts were collected from the roots. Root colonization expressed as log cfu/mg root after dilution plating of roots on solid media after 14 days of germination. The highest root colonization was reported by strains CLS2, CLS13, and CLS6, respectively, and these isolates were statistically similar and shared the same status. However, all the tested rhizobacterial strains were well capable of colonizing canola roots (Table 4). Biofilm was produced in the form of rings in the glass tubes. All these biofilm-forming strains synthesized rough-surfaced biofilms. The strains CLS13, CLS2, and CLS6 formed particularly rigid and thick biofilms, and the adjacent bacterial cells were not disseminated when the aggregates of these strains were agitated. Furthermore, the biofilms generated by CLS9 and CLS12 were thicker and more dense than those produced by CLS1 and CLS15. Conversely, CLS8 and CLS11 produced fragile and very thin biofilms, and these biofilms were easily spread when disturbed. The biomass biofilm varied significantly between the biofilm-producing strains. The highest amount of biomass biofilm was produced at OD_660_ by CLS2 (1.87 ± 0.05), followed by CLS6 (1.84 ± 0.07) and CLS13 (1.81 ± 0.04), which were statistically similar to each other, and followed by CLS9 (1.73 ± 0.04) and CLS12 (1.69 ± 0.05). The minimum quantity of biofilm biomass was built by CLS8 (1.39 ± 0.05). However, the biomass of biofilms differed not considerably between CLS1 (1.43 ± 0.07) and CLS15 (1.47 ± 0.06). CLS3 (1.63 ± 0.05) also produces a considerable amount of biofilm biomass (Table 4). Consequently, the quantity of biofilms and the bacterial numbers in these biofilms are affected by rhizobacterial strains.

**Table 4:** Quantitative screening of Canola (*Brassica napus* L.) associated zinc solubilizing rhizobacterial strains for different biochemical and Physiological attributes.

Isolates	Zinc solubilization (mgL^−1^ = ppm)	IAA production without L-tryptophan (μg ml^−1^)	IAA production with L-tryptophan (μg ml^−1^)	Nitrogen fixation (nmol h^−1^ mg^−1^)	Root colonization log cfu/mg root	Biofilm production biomass at (OD_660_)
CLS1	26.06 ± 0.95^bc^	8.92 ± 0.17^c^	10.56 ± 0.38 ^b^	71.03 ± 2.18 ^d^	6.98 ± 0. 14 ^ab^	1.43 ± 0.07 ^bcd^
CLS2	33.03 ± 0.84^a^	12.41 ± 0.41^ab^	16.62 ± 0.33^a^	124.58 ± 2.96^a^	7.13 ± 0.10^a^	1.87 ± 0.05^a^
CLS3	25.00 ± 0.86^c^	6.19 ± 0.15^d^	8.17 ± 0.47^c^	93.90 ± 2.25^c^	6.08 ± 0.18^c^	1.63 ± 0.05^abcd^
CLS6	32.43 ± 0.64 ^a^	12.98 ± 0.08^a^	16.94 ± 0.11^a^	123.44 ± 3.73^a^	7.12 ± 0.09^a^	1.84 ± 0.07^a^
CLS8	16.93 ± 0.95^d^	–	–	71.91 ± 3.57^d^	5.93 ± 0.19^cd^	1.39 ± 0.05^d^
CLS9	29.93 ± 0.86 ^ab^	11.74 ± 0.36^b^	15.57 ± 0.27^a^	106.35 ± 4.55^bc^	7.07 ± 0.11^ab^	1.73 ± 0.04^ab^
CLS11	17.40 ± 1.04^d^	–	–	70.49 ± 3.47^d^	5.92 ± 0.13^d^	1.41 ± 0.05^cd^
CLS12	29.46 ± 0.52 ^ab^	12.04 ± 0.18^ab^	15.84 ± 0.25^a^	104.56 ± 3.46^bc^	7.05 ± 0.17^ab^	1.69 ± 0.05^abc^
CLS13	32.63 ± 0.60^a^	12.90 ± 0.14^ab^	16.90 ± 0.15^a^	117.80 ± 1.94^ab^	7.13 ± 0.17^a^	1.81 ± 0.04^a^
CLS15	26.10 ± 0.96^bc^	8.65 ± 0.27^c^	10.63 ± 0.22^b^	73.33 ± 3.16^d^	6.94 ± 0.11^b^	1.47 ± 0.06^bcd^

Data presented here are the mean of three replications ± standard error; means sharing the same letters are statistically non significant and Tukey HSD test was performed at 5% (*p* ≤ 0.05) probability level.

### 3.5 Taxonomical identification of potential isolates using 16S rRNA gene amplification

**Figure 2:**
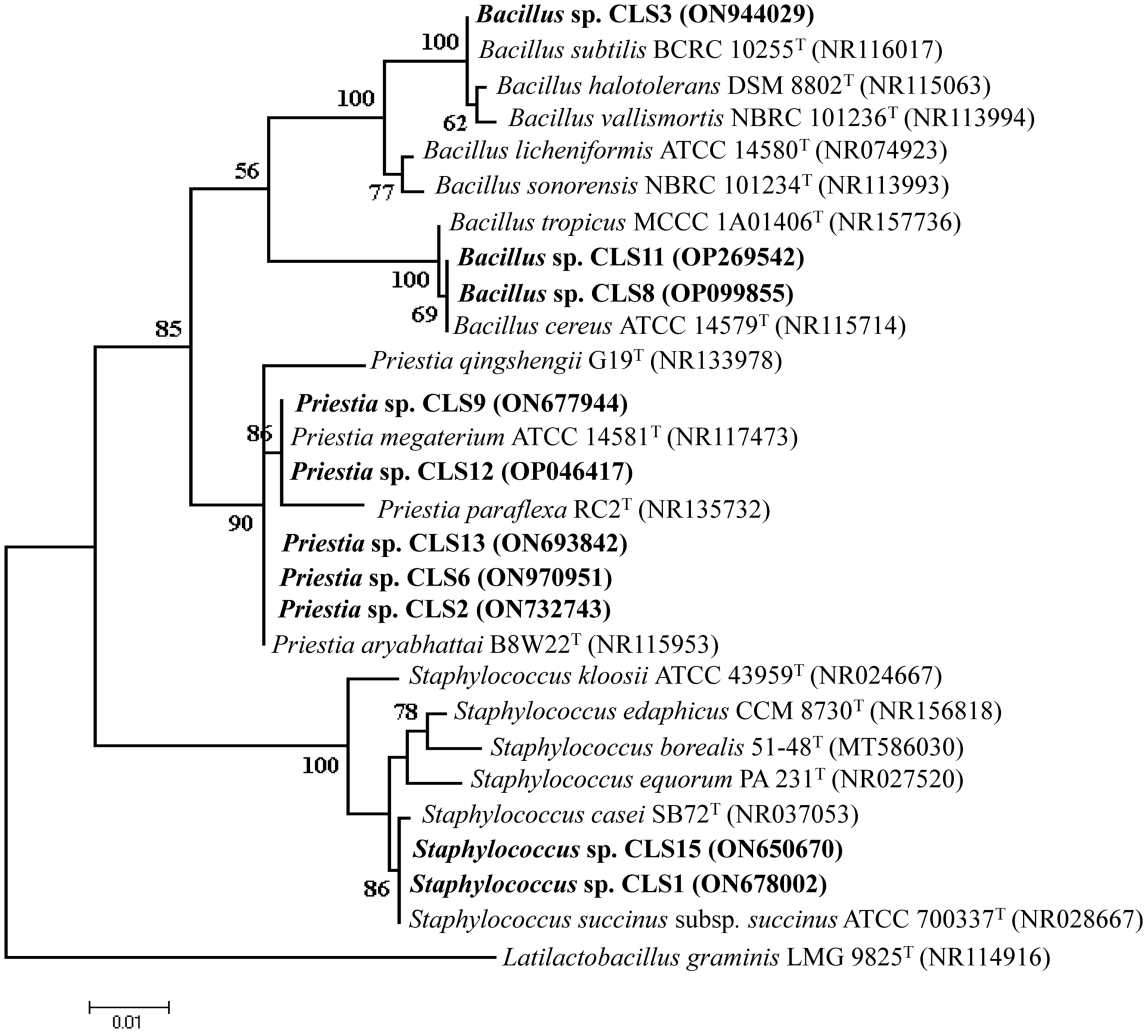
Maximum likelihood phylogenetic tree showing interrelationship of zinc-solubilizing strains (CLS1, CLS2, CLS3, CLS6, CLS8, CLS9, CLS11, CLS12, CLS13, CLS15) with closely related species of the genus Bacillus inferred from aligned sequences of the 16S rRNA gene. Tree was rooted by *Latilactobacillus graminis* (NR114916) as an outgroup. Bootstrap values expressed as a percentage of 1,000 replications are indicated at the nodes. The rhizobacterial isolates identified in the present study have been highlighted; accession number of each type strain is shown in brackets.

Genomic DNA from 10 potent rhizobacterial isolates was subjected to their 16S rRNA gene sequencing after PCR amplification. The 16S rDNA sequences of these isolates were compared with those of known 16S rRNA sequences. The identified sequences were deposited in GenBank, and their accession numbers and strains with maximum homology are given in Table 5. Phylogenetic analysis of the 16S rRNA gene indicated that 10 isolates belonged to different genera, *Bacillus*, *Staphylococcus*, and *Priestea*. Two isolates with the ability to solubilize zinc were *Staphylococcus* sp. CLS1 and CLS15, and these isolates formed cluster with *Staphylococcus succinus* subsp. *succinus* ATCC 700337^T^ in the phylogenetic tree (Figure 2). The most potent zinc-solubilizing isolates, CLS2, CLS6, and CLS13, were found to be phylogenetically similar to type strain *Priestia aryabhattai* B8W22^T^, showing 98.06–99.85% similarity in their 16S rRNA sequences (Table 5). The sequence analysis of the zinc-solubilizing isolates of CLS9 and CLS12 showed 98.78–99.03% homology with type strain *Priestia megaterium* strain B8W22^T^. Three isolates belonged to the genus *Bacillus*, of which two isolates CLS8 and CLS11 clustered with *Bacillus cereus* ATCC 14579^T^, while the isolate CLS3 showed sequence homology with *Bacillus subtilis* BCRC 10255^T^.

**Table 5:** Identification of Canola (*Brassica napus* L.) associated zinc solubilizing rhizobacterial strains based on 16S rRNA gene sequence analysis.

Rhizobacterial isolates	NCBI strains	Similarity	Gen Bank Accession No.
CLS1	*Staphylococcus succinus* NR028667	98.66%	ON678002
CLS2	*Priestia aryabhattai* NR115953	99.34%	ON732743
CLS3	*Bacillus subtilis* NR116017	99.73%	ON944029
CLS6	*Priestia aryabhattai* NR115953	98.06%	ON970951
CLS8	*Bacillus cereus* NR115714	99.50%	OP099855
CLS9	*Priestia megaterium* NR117473	99.03%	ON677944
CLS11	*Bacillus cereus* NR115714	99.68%	OP269542
CLS12	*Priestia megaterium* NR117473	98.78%	OP046417
CLS13	*Priestia aryabhattai* NR115953	99.85%	ON693842
CLS15	*Staphylococcus succinus* NR028667	99.65%	ON650670

### 3.6 Pathogenicity test

**Figure 3:**
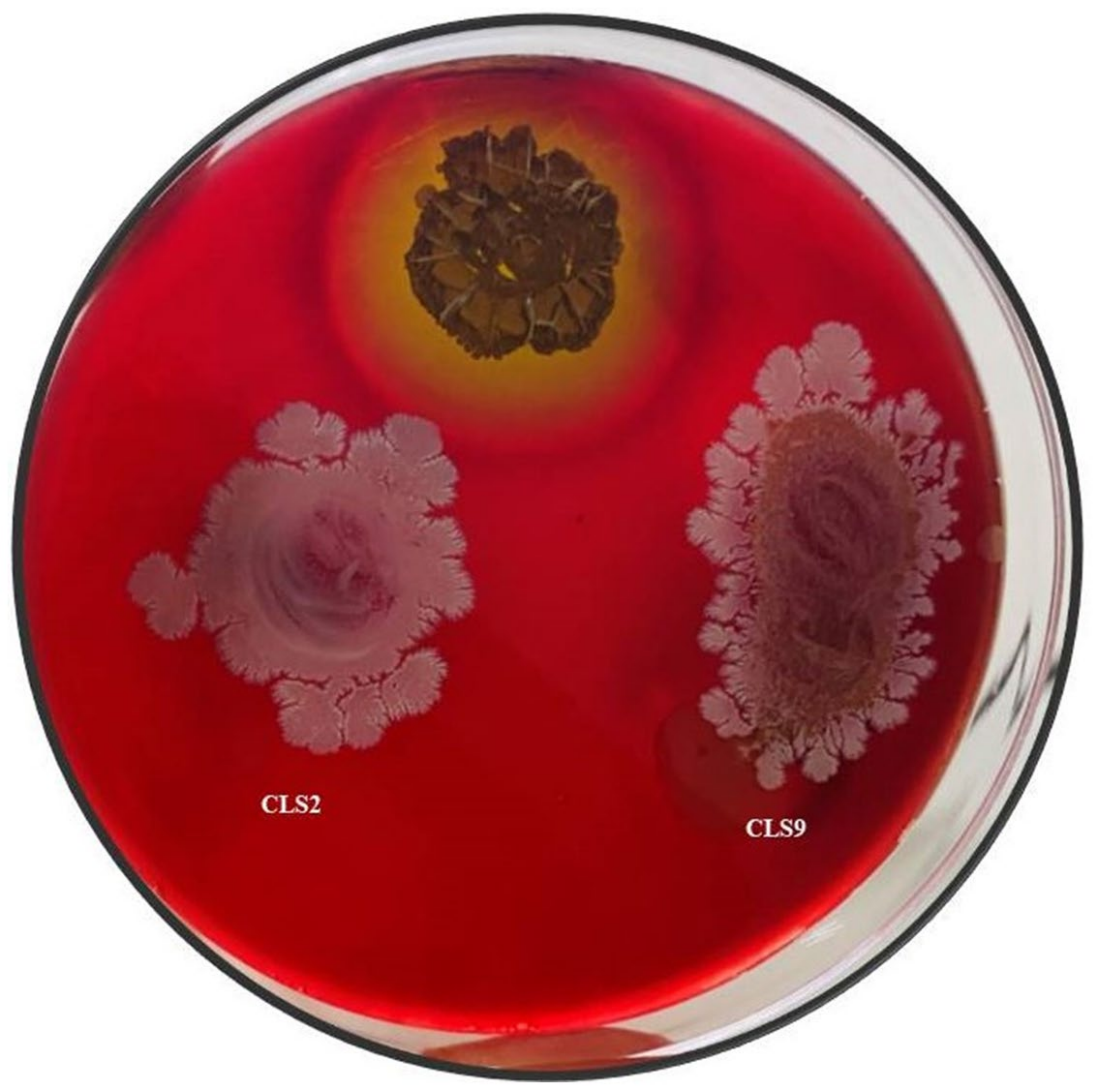
Selected rhizobacterial strains for pot and field trials showed growth on blood agar and their non-hemolytic nature as compared to the positive control (*Streptococcus pyogenes*).

A blood agar assay was performed to test the isolates for their biosafety. All of the rhizobacterial isolates used for the plant experiment were confirmed to be non-pathogenic due to the lack of hemolytic activity, verifying their safe use in future studies in field experiments. The results were recorded for the appearance of clear *β*, greenish-brown *α*, and no zones *γ*. Positive control *Streptococcus pyogenes* strain was used for comparison (Figure 3). Clear *β* zone on the blood agar plate was considered as a positive result.

### 3.7 Pot trial for testing zinc-solubilizing rhizobacterial strains for canola growth promotion

**Figure 4:**
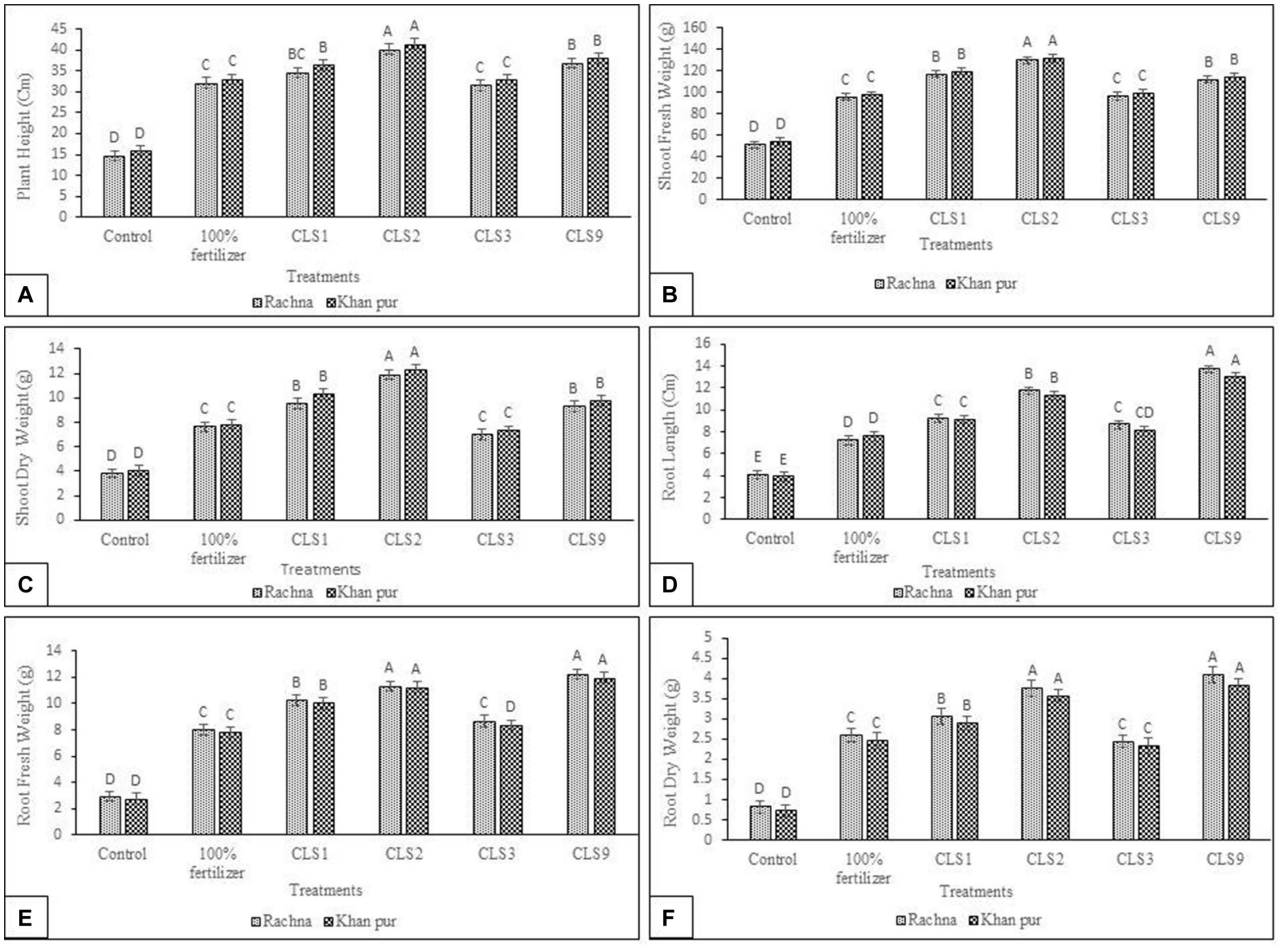
**(A–F)**: Effect of zinc-solubilizing isolates on promotion of growth attributes **(A)** Plant Height **(B)** Shoot Fresh Weight **(C)** Shoot Dry Weight **(D)** Root Length **(E)** Root Fresh Weight **(F)** Root Dry Weight of Canola (*Brassica napus* L.) cultivars (V1 Rachna; V2 Khan Pur) in Pot trial. Data were collected at the flowering stage. The Tukey HSD test was applied at a 5% (*p* < 0.05) probability level to determine significant differences between the means of the different treatments; the same letters on the bars indicate non-significant differences among the means, and vice versa.

In the present study, the potential of zinc-solubilizing rhizobacterial isolates *in vitro* to enhance canola growth in a pot experiment was tested. Seeds of two cultivars (V1 and V2) were inoculated with specified rhizobacterial strains capable of solubilizing zinc, which significantly increased canola growth. Seed priming with Zn-solubilizing isolates significantly increases the plant height, length of root, root and shoot fresh weight, root and shoot dry weight, and is statistically better than (without inoculation) control. The strain CLS2 was the best Zn solubilizer and showed a significant increase in plant height after that CLS9, which resulted in the highest increase of 2.72- and 2.50-fold, respectively, in plant height compared to control (non-inoculated) in cultivar V1. For V1, inoculation of CLS1 and CLS3 showed a notable increase in plant height by 2.36- and 2.14-fold, respectively, over non-inoculated control (Figure 4A). Comparison of treatment means revealed that maximum plant height was obtained in strain CLS2, which was followed by CLS9 and CLS1, statistically non-significant with each other in cultivar V2. Minimum plant height was recorded in non-inoculated control for cultivar V2. Whereas maximum increase of 2.53-fold in fresh weight of the shoot resulted with bacterization of CLS2, followed by CLS1 and CLS9 (2.28- and 2.18-fold, respectively) over non-inoculated control for V1 (Figure 4B). An increase of 2.14- and 1.88-fold in plant height and fresh weight of shoot, accordingly, was recorded by strain CLS3 and was non-significant to non-inoculated 100% fertilizer control. In cultivar V2, maximum increase of 2.44-fold in fresh weight of the shoot was noticed in CLS2. In case of shoot dry weight, maximum increase of 3.08-fold was obtained with the inoculation of isolate CLS2, followed by CLS1 and CLS9 (2.47- and 2.40-fold, respectively), which did not differ significantly from each other and were succeeded by CLS3, which was non-significant with non-inoculated 100% fertilizer control in cultivar V1 (Figure 4C). Results showed that priming of Zn-solubilizing isolates significantly promoted the root length, root fresh weight, and root dry weight over non-inoculated control. Highest increase in length of root, i.e., 3.38-fold, was noted by isolate CLS9 as compared to non-inoculated control (Figure 4D). Highest fresh weight of the root was acquired with the bacterization of CLS9, which enhanced fresh weight of the root by 4.18-fold over non-inoculated control. Isolate CLS3 responded efficiently by increasing fresh weight of the root by 2.95 times, but it was non-significant to 100% fertilize treatment (Figure 4E). Inoculation of CLS1, CLS2, and CLS3 enhanced the root dry weight by 3.69-, 4.53-, and 2.92-fold, respectively, compared to control (non-inoculated). The performance of all the zinc-solubilizing rhizobacterial strains was remarkable, but in comparison between these strains, the little increase in length of root, root fresh weight, and root dry weight was reported by the inoculation of CLS3 as compared to non-inoculated control. Significant rise in root dry weight was noted by 4.93-fold with the bacterization of CLS9 over control. The isolates that solubilized Zn showed their ability to enhance total dry weight and total fresh weight of canola plants. The pot experimental results disclosed that CLS2 inoculation was significantly better among all the isolates in terms of plant height and fresh and dry weight of shoots of canola cultivars V1 and V2. Moreover, the inoculation of CLS9 proved the most effective isolates in enhancing the length of root, root fresh, and dry weight of canola in both cultivars over non-inoculated control. Lowest growth attributes in relative plant height, length of root, fresh and dry weight of shoot, and fresh and dry weight of root were observed in non-inoculated control. The results showed that both cultivars of canola were different in their response to inoculation. Cultivar V2 showed better results in inoculation.

### 3.8 Field trial for testing zinc-solubilizing rhizobacterial strains for canola growth and yield

**Figure 5:**
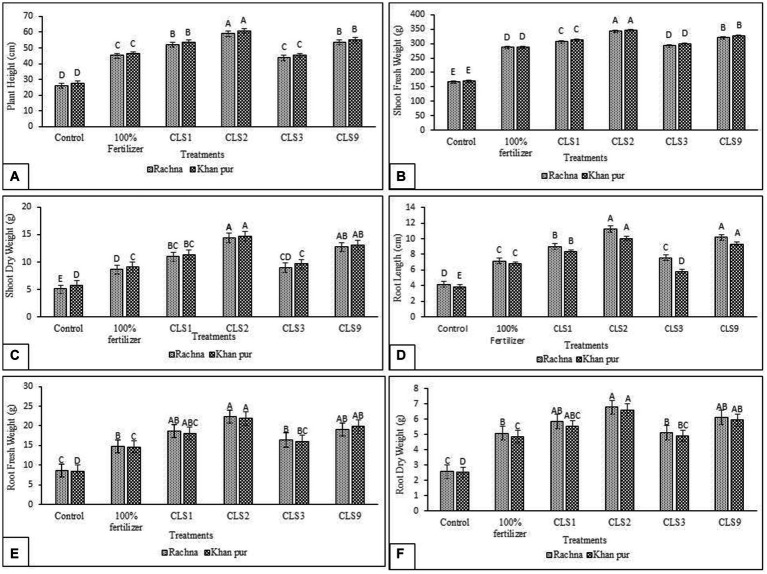
**(A–F)**: Effect of zinc-solubilizing isolates on promotion of growth attributes **(A)** Plant Height **(B)** Shoot Fresh Weight **(C)** Shoot Dry Weight **(D)** Root Length **(E)** Root Fresh Weight **(F)** Root Dry Weight of Canola (*Brassica napus* L.) cultivars (V1 Rachna; V2 Khan Pur) in the field trial. Data were collected at the flowering stage. The Tukey HSD test was applied at a 5% (*p* < 0.05) probability level to determine significant differences between the means of the different treatments; the same letters on the bars indicate non-significant differences among the means, and vice versa.

The selected promising zinc rhizobacterial strains were tested to investigate their impact on growth attributes of canola cultivars, i.e., Rachna (V1) and Khan pur (V2). The field soil was sandy loam in texture and alkaline in nature, having a pH of 8.2; EC of 2.6 dS m^−1^; 0.65% organic carbon; 0.047% N, 4.7 mg kg^−1^, and 197 ppm of available P and K, respectively. Total zinc concentration in the soil was 28.7 mg kg^−1^ and the available zinc concentration is 2.4 mg kg^−1^. In cultivar V1, strain *P. aryabhattai* (CLS2) displayed the highest increase (2.26-fold) of plant height over non-inoculated control (Figure 5A). The strains *P. megaterium* (CLS9) and *S. succinus* (CLS1) also showed a significant increase in plant height (2.06- and 2.00-fold) and were statistically non-significant with each other. The strain *B. subtilis* (CLS3) increased plant height by 1.68-fold when compared to non-inoculated control and was statistically similar with 100% fertilizer treatment. In cultivar V2, maximum plant height was observed in strain CLS2 with an increase of 2.23-fold over non-inoculated control, and in order of significance, CLS2 was followed by CLS9 and CLS1, which differed non-significantly with each other. In case of plant height, the strain CLS3 varied non-significantly with 100% fertilizer treatment in cultivar V2. Both cultivars showed non-significant differences between treatment means in the case of plant height of canola. However, the cultivar means showed that V2 was significantly better than V1. A maximum (2.06-fold) increase in fresh weight of shoot over non-inoculated control was observed from CLS2, followed by CLS9 and CLS1, which reported 1.92- and 1.84-fold higher over non-inoculated control in V1 (Figure 5B). In case of inoculation minimum shoot fresh weight was recorded in CLS3 with an increase of 1.76-fold, which was statistically similar to 100% fertilizer treatment in V1.

All treatments showed the same pattern of growth in plant height and fresh weight of shoot and showed the statistically same response to inoculation in both cultivars (V1 and V2). The cultivar V2 proved better in shoot dry weight and statistically significant to V1. Maximum shoot dry weight was observed in CLS2 in both cultivars with a rise up to 2.83- and 2.55-fold compared to non-inoculated control in cultivars V1 and V2, respectively. Isolate CLS9 showed the second-best result in shoot dry weight, followed by CLS1 and CLS3, while CLS3 was almost at par with 100% fertilizer treatment in both cultivars (V1 and V2). The results showed that both cultivars are non-significant with each other in shoot dry weight (Figure 5C). The strain CLS2 recorded a maximum growth of 2.67- and 2.57-fold in root length when compared to non-inoculated control in cultivars V1 and V2, respectively (Figure 5D) and was statistically non-significant to strain CLS9 in both cultivars. CLS1 also reported a better increase of 2.15- and 2.14-fold in root length over non-inoculated control in cultivars V1 and V2, respectively. The strain CLS3 was found less effective among the other inoculating zinc-solubilizing strains, but it was non-significant to 100% fertilizer treatments and significantly higher than non-inoculated control in cultivar V1. However, in cultivar V2, the strain CLS3 was less significant to 100% fertilizer treatments but statistically significant to non-inoculated control. The highest fresh weight of root in cultivar V1 was recorded from strain CLS2, with a rise of 2.60-fold over non-inoculated control, followed by CLS9 and CLS1, which were statistically similar to each other, increasing up to 2.22- and 2.16-fold, respectively, compared to non-inoculated control. Strain CLS3 was also better at producing root fresh weight, increasing to 1.90-fold as compared to non-inoculated control and non-significant with non-inoculated 100% fertilizer treatment in cultivar V1 (Figure 5E). In case of V2, maximum root fresh weight was obtained in CLS2, which was followed by strain CLS9 and CLS1, with an increase up to 2.36- and 2.16-fold, respectively, over non-inoculated control. Minimum values of root dry weight among the inoculated strain treatments were observed in CLS3, but it varied significantly with non-inoculated 100% fertilizer treatment as well as non-inoculated control. Both cultivars were non-significant with each other in root fresh and dry weight of canola. In cultivar V1, the inoculated plants with strain CLS2 showed a tremendous increase in root dry weight (2.61-fold) compared to non-inoculated control. The isolates CLS9 and CLS1 showed significant increase up to 2.35- and 2.25-fold in root dry weight as compared to non-inoculated control respectively, and were statistically non-significant with each other in cultivar V1 (Figure 5F). In the case of inoculation, the minimum root dry weight was shown by the strain CLS3 with an increase of 1.97-fold over non-inoculated control and statistically similar to the non-inoculated 100% fertilizer-treated plants in cultivar V1. In cultivar V2, maximum root dry weight was observed in strain CLS2, with an increase of 2.62-fold over non-inoculated control. The strain CLS9 showed the second-best result with 2.35-fold increase in dry weight of root over non-inoculated control, followed by strains CLS1 and CLS3, which were significantly higher than non-inoculated 100% fertilizer treatment and non-inoculated control as well. No significant variation in root dry weight occurred between cultivars V1 and V2 of canola. Overall, the findings of the field trials demonstrated the beneficial effects of *P. aryabhattai* (strain CLS2) and *P. megaterium* (strain CLS9) on both cultivars of canola plant. In assessment of the total effect of these bacterial inoculants, strain CLS2 was the best and manifested tremendous increase in all growth attributes, plant height, root length, fresh and dry weight of shoot, and fresh and dry weight of root. The isolate CLS9 was the second-best strain, showing remarkable growth in all growth attributes enlisted above. While non-inoculated control reported minimum plant height, length of root, fresh and dry weight of shoot, and fresh and dry weight of root. The effect of the seed-applied zinc strains on growth traits was displayed in Figures 5A–F. However, in both sand- (Pot) and soil (Field)-based experiments, CLS2 displayed the highest and most consistent rise in plant height, dry and fresh weight of shoot, and fresh and dry weight of root, followed by CLS9, and then CLS1. CLS2 displayed the highest and most consistent rise in plant height, shoot dry, and, however, strain *B. subtilis* (CLS3) was found less effective among the other inoculating zinc-solubilizing strains, but it was significantly higher than non-inoculated control in all growth attributes of canola. In the field trial investigations, the *P. aryabhattai* (CLS2) strain was the best strain for increasing canola plant growth in terms of total plant dry weight.

### 3.9 Potential of rhizobacterial strains on yield attributes of canola

**Figure 6:**
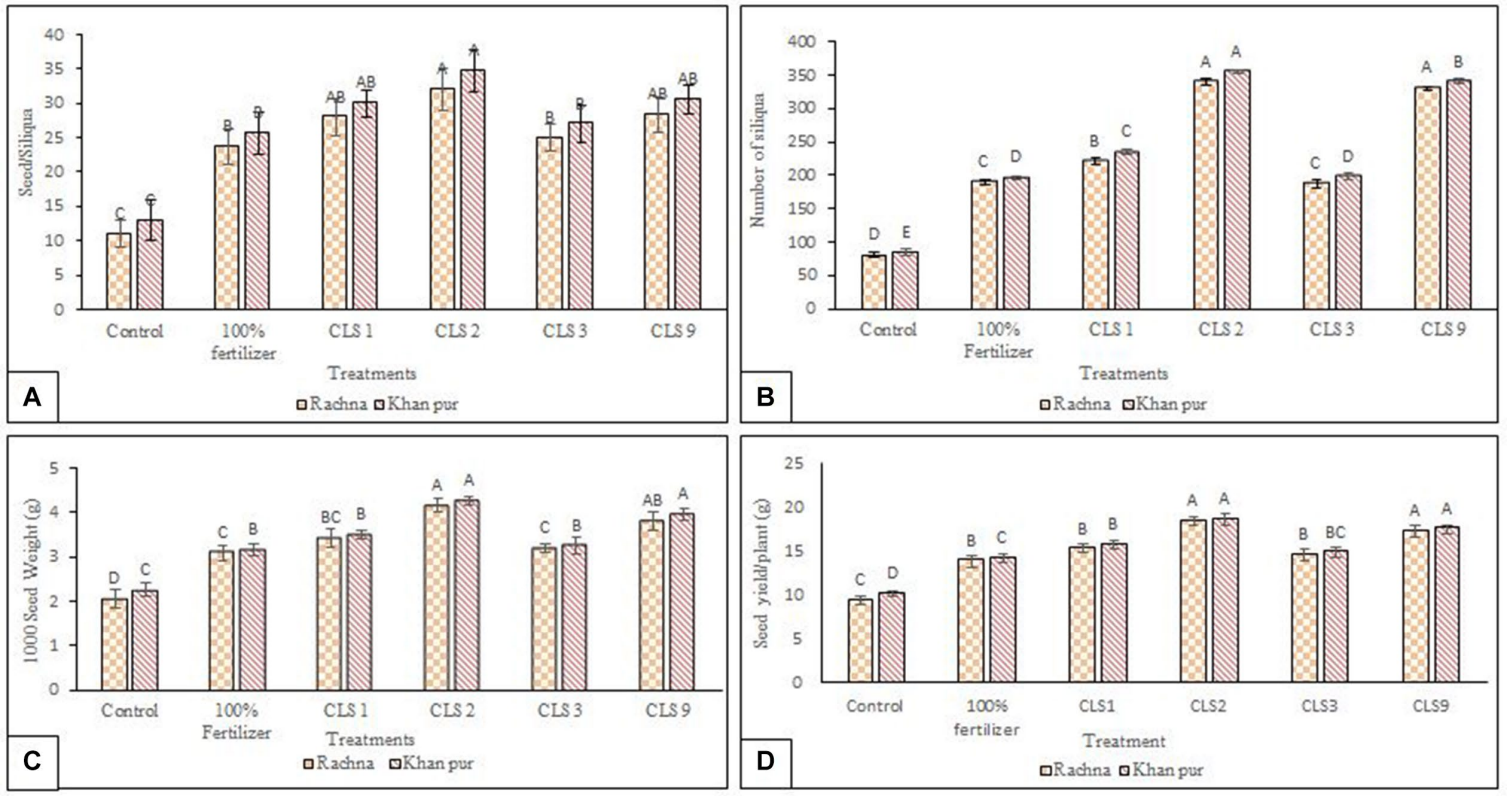
**(A–D)**: Effect of zinc-solubilizing rhizobacteria on yield components of canola cultivars V1 (Rachna) and V2 (Khan Pur); **(A)** number of seeds/siliqua and **(B)** number of silique/plant **(C)** 1,000 seed weight and **(D)** seed yield/plant. Data for yield attributes were collected at the harvesting stage. The Tukey HSD test was applied at a 5% (*p* < 0.05) probability level to determine significant differences between the means of the different treatments; the same letters on the bars indicate non-significant differences among the means, and vice versa.

Data pertaining to the yield attributes of canola cultivars as affected by different zinc-solubilizing isolates have been given in Figures 6A–D. The strain CLS2 demonstrated highest number of silique/plant with a 4.19- and 4.21-fold increase compared to the non-inoculated control in both cultivars. The strain CLS2 was statistically similar to strain CLS9, which had an increase of 4.08-fold in the number of siliqua of plant as compared to non-inoculated control in V1 (Figure 6A). Strain CLS1 showed remarkable increase (2.72- and 2.78-fold) in number of siliqua/plant in cultivars V1 and V2, respectively. In both cultivars, strain CLS3 was statistically identical to non-inoculated 100% fertilizer treatment, but these treatments were statistically significant from non-inoculated control in the number of siliqua/plant. On comparison of cultivars, it was revealed that higher number of siliqua was observed in cultivar V2 than V1. In cultivar V1, strain CLS2 reported a significant maximum number of seeds/silique with a 2.90-fold increase, followed by strains CLS9 and CLS1 having a 2.57- and 2.54-fold increase, respectively, over non-inoculated control and these strains were statistically similar with each other. Strain CLS3 was statistically non-significant to non-inoculated 100% fertilizer treatment, but these treatments were statistically different from non-inoculated control in cultivar V1 (Figure 6B). In cultivar V2, a comparative view of treatment means indicated that maximum increase (2.66-fold) of seeds/silique over non-inoculated control was observed in strain CLS2, followed by CLS9 and CLS1, which were non-significant with each other but significantly higher than non-inoculated control. Among the inoculation treatments, minimum values of seeds/silique were recorded by strain CLS3 varied non-significantly with non-inoculated 100% fertilizer treatment but statistically significant to non-inoculated control. All the treatment applications in the case of seeds/silique of canola showed statistically similar responses for both cultivars of canola; however, the cultivar means showed that V2 was significantly better than V1. In cultivars V1 and V2, the highest increase of 2.01 and 1.88, respectively, fold in 1,000 seed weight was recorded due to the application with strain CLS2 compared to the non-inoculated control (Figure 6C). The strain CLS2, followed by CLS9 and CLS1. These strains were statistically similar to each other up to an increase of 1.84- and 1.66-fold in 1,000 seed weight in V1. The strain CLS3 was statistically similar to non-inoculated 100% fertilizer treatment in both cultivars. Similar response of increase in 1,000 seed weight was noted by all the treatments in both cultivars; however, cultivar means indicated that V2 was significantly higher than V1. Strain CLS2 showed a maximum seed yield/plant with an increase of 1.97- and 1.83-fold over non-inoculated control in V1 and V2, respectively, and was statistically similar to strain CLS9 in both cultivars. In case of seed yield/plant, the strains CLS9, followed by CLS1 and CLS3, which were statistically similar to each other and with non-inoculated 100% fertilizer treatment but varied significantly to non-inoculated control in V1 cultivar. The strain CLS1 caused an increase of 1.54-fold in seed yield/plant, followed by CLS3, which was statistically significant to non-inoculated 100% fertilizer treatment and non-inoculated control as well in V2 (Figure 6D). The yield of canola crop was significantly enhanced by seed priming with zinc-solubilizing rhizobacteria, while the number of seeds/siliqua, number of siliqua/plant, 1,000 seed weight, and seed yield/plant increased significantly with rhizobacterial treatment compared to non-inoculated control at harvest stage. Non-inoculated control reported minimum values for all the yield parameters. However, cultivar V2 was significantly better than V1 within all the yield parameters.

### 3.10 Effect of zinc-solubilizing isolates on oil content and fatty acid profile

Variable results of oil, protein contents, and fatty acid profile as affected by zinc-solubilizing strains among both cultivars are illustrated in Tables 6, 7. Inoculation of rhizobacterial strains significantly enhanced the oil contents of the seeds of canola. Our results indicated the significant differences between non-inoculated and inoculated seeds with rhizobacterial strains on oil and protein content. Oil content increased with non-inoculated control with 100% fertilizer. The highest oil content was observed in CLS2 and CLS9, but there was no statistical difference between the oil contents of the seeds treated with CLS2 and CLS9. The lowest oil contents belong to non-inoculated seeds (without fertilizer), followed by non-inoculated seeds with 100% fertilizer. On the other hand, linoleic acid was decreased in the following manner non-inoculated control (with 100% fertilizer) > CLS9 > CLS3 > CLS2 in contrast with non-inoculated control (without fertilizer). Just like linoleic acid, bacterial inoculation imposed a significant positive impact on linolenic acid in Table 6. Variable impact of linolenic acid in canola seeds was observed among all the inoculated treatments, where highest increase was recorded in CLS2-treated seeds in both varieties, followed by non-inoculated control (with 100% fertilizer) and CLS9, which were non-significant with each other but differed significantly to non-inoculated control. The lowest value of linolenic acid was recorded in non-inoculated control for both varieties. Highest increase in palmitic acid was observed in non-inoculated control seeds, followed by CLS1 and CLS3. However, CLS2 differed non-significantly to the CLS9 compared to the non-treated control. Bacterial inoculation imposed non-significant impact on stearic acid in variety V1 compared to non-treated control. However, in V2, highest value of stearic acid was found in seed treated with CLS3, which was non-significant with non-treated control (with 100% fertilizer), followed by CLS1, CLS9, and non-treated control, which were also non-significant with each other. Ecosenoic acid was observed to be maximum for CLS2 treated seeds, followed by CLS1, CLS3, CLS9, and non-inoculated control with 100% fertilizer, which were non-significant with each other but significantly differed with inoculated control. However, the effect of inoculated treatments on ecosenoic acid in seeds of V2 remained non-significant. The values of glucosinolates were significantly high in non-inoculated control with 100% fertilizer. Maximum values of glucosinolates were recorded in seeds treated with 100% fertilizer followed by bacterial inoculation CLS3, which was non-significant with control. Lowest values of glucosinolates were observed in seeds treated with CLS9. Both varieties displayed the same effect of all the treatments on glucosinolate accumulation. Erucic acid in the seeds of canola was substantially decreased among all the inoculated treatments as compared with the untreated control with and without fertilizer. The fatty acid profile of canola seeds of both cultivars shown in Table 7 revealed that erucic acid was determined to be 0.56–0.63% and glucosinolates 17.77–52.19 μmol g^−1^.

**Table 6:** Effect of zinc solubilizing isolates on oil content and fatty acid profile in seeds of Canola (*Brassica napus* L.) cultivars in field trial.

	Oil contents (%)		Protein (%)		Oleic acid (%)		Linoleic acid (%)		Linolenic acid (%)	
Canola cultivars	V1	V2	V1	V2	V1	V2	V1	V2	V1	V2
Uninoculated control	26.89 ± 0.45^d^	27.75 ± 0.29^d^	20.92 ± 0.39^a^	20.60 ± 0.48^a^	40.12 ± 0.23^d^	41.22 ± 0.37^e^	13.82 ± 0.59^d^	12.90 ± 0.40^d^	4.57 ± 0.27^c^	4.72 ± 0.34^d^
100% fertilizer	35.98 ± 0.29^c^	36.99 ± 0.44^c^	19.64 ± 0.37^ab^	16.57 ± 0.62^b^	47.94 ± 0.43^c^	49.81 ± 0.49^cd^	20.86 ± 0.39^a^	20.63 ± 0.64^a^	6.26 ± 0.14^ab^	6.50 ± 0.24^abc^
*Staphylococcus succinus* (CLS1)	38.67 ± 0.65^b^	39.43 ± 0.63^b^	18.34 ± 0.71^bcd^	18.47 ± 0.69^ab^	51.43 ± 0.51^b^	52.30 ± 0.57^bc^	14.35 ± 0.19^cd^	14.07 ± 0.13^cd^	4.60 ± 0.32^c^	4.83 ± 0.33^cd^
*Priestia aryabhattai* (CLS2)	42.97 ± 0.05^a^	44.07 ± 0.30^a^	17.02 ± 0.88^cd^	16.91 ± 0.20^b^	54.35 ± 0.68^a^	56.02 ± 0.92^a^	15.11 ± 0.38^cd^	15.00 ± 0.37^bc^	6.68 ± 0.25^a^	6.91 ± 0.19^a^
*Bacillus subtilis* (CLS3)	38.87 ± 0.49^b^	40.18 ± 0.46^c^	18.81 ± 0.14^abc^	18.19 ± 0.68^ab^	48.81 ± 0.44^c^	49.08 ± 0.90^d^	16.01 ± 0.21^bc^	15.70 ± 0.29^bc^	4.97 ± 0.39^bc^	5.18 ± 0.47^bcd^
*Priestia megaterium* (CLS9)	42.09 ± 0.30^a^	42.92 ± 0.42^a^	15.92 ± 0.31^d^	17.17 ± 0.40^b^	53.26 ± 0.79^ab^	53.69 ± 0.56^ab^	17.00 ± 0.40^b^	16.49 ± 0.38^b^	6.28 ± 0.48^ab^	6.70 ± 0.45^ab^
Cultivar means	37.58^b^	38.56^a^	18.44^a^	17.98^a^	49.32^b^	50.35^a^	16.19^a^	15.80^a^	5.56^a^	5.81^a^

Effect of zinc solubilizing rhizobacteria on oil contents and fatty acid profile of canola cultivars presented in this table are the three replications ± standard error; Canola cultivars are V1 (Rachna) and V2 (Khan Pur). Data for measurement of fatty acid profile of seeds was collected at maturity of canola crop. The Tukey HSD test was applied at a 5% (*p* ≤ 0.05) probability level to determine significant differences between the means of the different treatments; the same letters on the bars indicate non-significant differences among the means, and vice versa; CVC, critical value for comparison.

**Table 7:** Effect of zinc solubilizing isolates on oil content and fatty acid profile in seeds of Canola (*Brassica napus* L.) cultivars in field trial.

	Palmitic acid (%)		Stearic acid (%)		Ecosenoic acid (%)		Erucic acid (%)		Glucosinolates (μmol/g)	
Canola cultivars	V1	V2	V1	V2	V1	V2	V1	V2	V1	V2
Uninoculated control	6.23 ± 0.18^a^	6.03 ± 0.18^a^	2.28 ± 0.09^a^	2.45 ± 0.13^ab^	3.98 ± 0.22^b^	4.29 ± 0.21^a^	2.64 ± 0.26^a^	2.58 ± 0.18^a^	33.62 ± 0.76^b^	33.10 ± 0.64^b^
100% fertilizer	5.77 ± 0.22^ab^	5.66 ± 0.18^a^	2.43 ± 0.16^a^	2.62 ± 0.21^a^	4.31 ± 0.18^ab^	4.45 ± 0.21^a^	1.35 ± 0.11^b^	1.25 ± 0.07^b^	52.19 ± 1.34^a^	50.75 ± 1.09^a^
*Staphylococcus succinus* (CLS1)	5.66 ± 0.25^ab^	5.40 ± 0.17^ab^	2.09 ± 0.32^a^	2.28 ± 0.21^ab^	4.03 ± 0.38^ab^	4.19 ± 0.40^a^	0.73 ± 0.04^bc^	0.63 ± 0.02^c^	22.98 ± 0.94^c^	23.90 ± 0.70^c^
*Priestia aryabhattai* (CLS2)	4.57 ± 0.19^c^	4.36 ± 0.16^b^	1.61 ± 0.05^a^	1.73 ± 0.04^b^	5.52 ± 0.35^a^	5.71 ± 0.32^a^	0.71 ± 0.08^c^	0.56 ± 0.08^c^	23.61 ± 1.17^c^	25.10 ± 1.24^c^
*Bacillus subtilis* (CLS3)	4.81 ± 0.30^bc^	5.07 ± 0.37^ab^	2.40 ± 0.18^a^	2.60 ± 0.15^a^	4.20 ± 0.23^ab^	4.39 ± 0.23^a^	0.87 ± 0.04^bc^	0.84 ± 0.04^bc^	29.90 ± 0.96^b^	30.92 ± 1.12^b^
*Priestia megaterium* (CLS9)	4.23 ± 0.11^c^	4.38 ± 0.15^b^	2.02 ± 0.10^a^	2.19 ± 0.15^ab^	4.39 ± 0.43^ab^	4.59 ± 0.49^a^	0.79 ± 0.08^bc^	0.63 ± 0.09^c^	17.77 ± 0.68^d^	18.45 ± 0.32^d^
Cultivar means	5.21^a^	5.15^a^	2.14^a^	2.31^a^	4.40^a^	4.60^a^	1.18^a^	1.08^a^	30.01^a^	30.37^a^

Effect of zinc solubilizing rhizobacteria on fatty acid profile of canola cultivars presented in this table are the three replications ± standard error; Canola cultivars are V1 (Rachna) and V2 (Khan Pur). Data for measurement of fatty acid profile of seeds was collected at maturity of canola crop. To calculate significant differences among the means of different treatments, Tukey HSD test was applied at 5% (*p* ≤ 0.05) probability level. The same letters on the bars show the non-significant differences between the mean, and vice versa.

## 4 Discussion

Keeping in view the role of zinc-solubilizing rhizobacterial isolates on the growth and production of canola plants, the present study was aimed to isolate and identify the promising strains which were potential candidates to solubilize zinc and other nutrients. Ten isolated Zn-solubilizing bacteria associated with canola roots, rhizosphere, and soil were cataloged from 84 samples, and the identification of these rhizobacterial strains was used to verify the diversity of isolates in different sampling sites. Overall, our results indicated that bacteria belonging to the *Bacillus* genera were the dominant root-associated bacteria of canola, identified as *B. cereus*, *B. subtilus*, *P. aryabhattai*, and *P. megaterium* from the canola rhizosphere of all three locations. *P. aryabhattai* was the dominant strain in all the rhizospheric samples collected from three different locations. *Priestia* members were originally categorized as *Bacillus* species, a genus having extensive polyphyly among its members. *P. megaterium* also showed good association with canola plants that were isolated from two locations. While the strain *S. succinus* was also isolated from two sites. In our study, similar isolates identified from three different sampling sites of canola plants indicated that these strains were host-specific. Our results are similar to the findings of Rani et al. (2023) who also found that the crop-specific Zn-solubilizing bacteria from several locations throughout the globe. This research is an update about the role of *Bacillus* and *Staphylococcus* spp. as zinc solubilizers in canola crops as inexpensive economic agricultural inputs. At different sampling sites, the availability of same bacterial strains associated with host plants (canola) is highly valuable for planning future investigations due to the important role of these rhizospheric bacteria in oil seed crop enhancement studies. Zinc is one of the most requisite micronutrients required for effective growth and development of plants. It ameliorates canola productivity in addition to providing nutritional security. Its deficiency not only affects nutritional quality, but also growth and yield of canola. Since Pakistani soil is alkaline and calcareous in nature, the pH is found around 6.8–9.2 (Samreen et al., 2019). Due to high pH and low organic matter of Pakistani soil, it is zinc-deficient. Soil-applied inorganic zinc becomes unavailable soon after its application. By decreasing the soil pH, zinc-solubilizing rhizobacteria secrete organic acids and transform the insoluble forms of inorganic zinc into plant-soluble forms of zinc, so it becomes available in soil for plant uptake. Similar findings have also been documented in previous work of Masood et al. (2022) and Ali et al. (2023). To date, little is known about interventions such as the use of soil bacteria to solubilize unavailable zinc and improve zinc uptake in plants. Its well-known zinc mobilization abilities appear to be prevalent among bacterial taxa, *Bacillus*, which is one of the most extensively researched genera because it is found to be abundant in nature and has many growth-enhancing characteristics (Zhao et al., 2011; Naseer et al., 2020). In our investigation, we identified rhizobacterial strains with unique characteristics, such as improved plant growth through mitigation of zinc. Among the identified strains, the genus *Bacillus* was the most dominant in terms of solubilizing non-labile zinc in soil and promoting canola growth, yield, and nutrient accumulation in grains. All of these Zn-solubilizing *Bacillus* strains are Gram-positive plant-associated bacteria that secrete growth-promoting metabolites that enhance plant growth, nutrient acquisition, and suppress soil-borne plant pathogens (Meena et al., 2017). Among 10 zinc-solubilizing strains, four zinc-solubilizing bacterial strains were screened *in vitro* for their plant growth-promoting abilities based on their Zn solubilization efficiency and Zn solubilization index. The zinc-solubilizing bacteria produce a variety of secondary metabolites that enhance Zn availability and plant growth (Yadav et al., 2022). The results are similar to those stated by Upadhyay et al. (2021), who found that inoculation of zinc-solubilizing bacteria notably increased plant height and root length of maize plants. Bacterial solubilization of Zn-mediated organic acid secretion may result in a drop in pH, which is important for enhancing their Zn solubility and uptake (Ramesh et al., 2014). Our findings were also supported by earlier reports, where increase in zinc availability was achieved by drop of pH of the medium (Mumtaz et al., 2019). Previously, various zinc-solubilizing *Bacillus* strains *viz. B. aryabhattai* (Siddikee et al., 2010; Mumtaz et al., 2017; Naseer et al., 2020), *B. subtilis* (Liu et al., 2018; Abbaszadeh-Dahaji et al., 2020; Ahmad et al., 2021), *B. cereus* (Khande et al., 2017; Mumtaz et al., 2019) and *B. megaterium* (Dinesh et al., 2018; Bhatt and Maheshwari, 2020; Rezaeiniko et al., 2022) were reported as ideal candidates for biofortification of Zn in several crops. Among these isolated strains, CLS2 showed a remarkable solubilization potential for zinc oxide by producing large clear halo zones and expressed highest potential as zinc solubilizer plant growth-promoting isolate throughout the pot and field experiments. Therefore, meticulous use of this isolated strain CLS2 could help in providing a considerable amount of soluble zinc along with augmented nutrient uptake, plant growth, and yield in sustainable manner (Bhatt and Maheshwari, 2020). Strain CLS2 produced siderophore, hydrogen cyanide, and protease, which was in line with the findings of (Mumtaz et al., 2017) who also declared the positive outcomes for similar characteristics in *B. aryabhattai* strains. The plant experiment results also show that canola seeds inoculated with *B. megaterium* (CLS9) performed second best among all other treatments, resulting in increased root and shoot length and fresh and dry weight of root and shoot. In our study, inoculation of *P. aryabhattai*, followed by *P. megaterium*, *S. succinus*, and *B. subtilis* performed better in terms of growth and production of canola. Castiglione et al. (2021) highlighted the plant growth-promoting features (IAA and siderophore production and solubilization of phosphate) of *S. succinus* in different crops (quinoa and maize), while Orhan and Demirci (2020) observed the ability of nitrogen fixation exhibited by *S. succinus*. According to Oliva et al. (2023), *S. succinus* promotes the production of fine roots and an increase in root fresh and dry weight of maize plants. However, to the best of our knowledge, this is the first report demonstrating the significance of *S. succinus* (CLS1) as an efficient zinc solubilizer, nutrient enhancer, and plant growth promoter isolated from canola rhizosphere. As a result, it is recommended as an alternative to chemical fertilizers. Maximum values for production of auxins, root colonization, and phosphate and zinc solubilization ability were found in these four selected strains, which could be used to improve canola growth and yield. The same results were presented by Pandey et al. (2018), who found that the *B. pumilus* and *B. subtilis* improved *Amaranthus* growth parameters. Previous reports also demonstrated that PGB inoculation increases the growth of canola (Nadeem et al., 2014). Increased canola growth could be related to the potential of Zn-solubilizing rhizobacterial isolates to increase nutrient availability through nitrogen fixation, solubilizing the insoluble phosphate, starch hydrolysis, zinc solubilization, and the production of phytohormones and siderophores. Several studies have been reported, where a range of microbial genera capable of solubilizing different zinc sources have been procured from canola (Dabrowska et al., 2017; Masood et al., 2022). In comparison to the non-inoculated control, the inoculation of Zn-solubilizing rhizobacterial isolates extensively improved growth parameters. Our results are also similar to those of Ramesh et al. (2014) who stated that inoculation of zinc-solubilizing strain *P. aryabhattai* increased the plant height, shoot, and root dry weight in soybeans and wheat. The second-best zinc solubilizer strain, *P. megaterium*, is a nutrient enhancer and plant growth promoter, suggesting that it may be employed as a substitute for nutritional shortfalls in the host plant by converting insoluble zinc to the soluble one and as an alternative to synthetic chemical fertilizers. Inoculation of *B. subtilis* as zinc-solubilizing rhizobacterial strain significantly increased dry matter and grain yield, which was followed by *B. megaterium* in soybean plant (Danhorn and Fuqua, 2007). Biofilm formation is crucial for efficient root colonization, provides the bacteria an advantage to compete with other microorganisms, and increases chances of bacterial survival in hostile environments (Ramey et al., 2004; Yuan et al., 2015; Altaf and Ahmad, 2016). *P. aryabhattai* (CLS2) showed the highest rate of ability to form biofilm. Therefore, biofilm production could be relevant to the isolates to assess their behavior in field trials. Biofilm formation is crucial for efficient root colonization, provides the bacteria an advantage to compete with other microorganisms, and increases chances of bacterial survival in hostile environments (Ramey et al., 2004; Yuan et al., 2015; Altaf and Ahmad, 2016). *P. aryabhattai* (CLS2) showed the highest rate of ability to form biofilm. Therefore, biofilm production could be relevant for the isolates to assess their behavior in field trials. In addition to this, our study goes well with Ansari et al. (2023), who also reported the multifunctional PGP traits as well as rhizosphere colonization and biofilm formation by *B. subtilis*, which enhanced the growth of wheat plants. Similar findings have also been documented in the previous work of Bach et al. (2022) who isolated *B. aryabhattai* strains from canola rhizosphere, and these strains formed the biofilm and showed the synergic action by the production of siderophore. Soil porosity and water contents were also influenced by rhizobacterial EPS secretions (Shahzad, 2020). Thus, production of EPS by microbial isolates in the plant rhizosphere stabilized the soil aggregates by improving porosity, root proliferation, water contents, and their gummy exudations (Deka et al., 2019; Khan and Bano, 2019; Cheng et al., 2020). To delay soil drying locally and related adverse effects, *Bacillus subtilis* and plants modify their surroundings by releasing EPS (Benard et al., 2023). Significant root colonization is required for rhizobacteria to develop themselves adequately in the rhizosphere, and PGP rhizobacteria are an effective way of enhancing agricultural output by suppressing plant pathogens (Zhou et al., 2016; Kalam et al., 2020; Jiao et al., 2021). Compared to other test isolates, CLS2 and CLS9 were stronger root colonizers. Our results also revealed that zinc-solubilizing isolates *B. megaterium* and *B. cereus* were also capable of solubilizing inorganic as well as organic phosphate. Furthermore, our findings are consistent with those of Dubey and Maheshwari (2011), who discovered that *B. subtilis* solubilizes zinc and promotes canola production. Previously, it was demonstrated that these Zn-solubilizing *Bacillus* strains had multiple plant growth-promoting characteristics and the ability to promote maize growth, yield, and nutrient uptake (Mumtaz et al., 2017; Mumtaz et al., 2018; Mumtaz et al., 2022). The Zn-solubilizing *Bacillus* strains have been well documented for the production of IAA, which plays an essential role in plant microbe associations that increase and promote crop growth and production (Saxena et al., 2020). Our results showed that all the tested isolates in pot and field experiments have the ability to produce IAA both in the presence and absence of L-tryptophan. Highest IAA produced by isolates CLS2 and CLS9 might play a role in enhancing the growth of canola plants. Our results are also in line with those of Iqbal et al. (2023) who also claimed the increased canola crop growth and yield with the introduction of IAA synthesizing *Bacillus* strains. Furthermore, a recent study on Zn-solubilizing bacterial strains was revealed to enhance rice crop growth parameters (Shakeel et al., 2023). Previously, Asghar et al. (2002) suggested the production of IAA by bacterial strains isolated from the rhizosphere of *Brassica* species to improve growth yield and oil content of canola. Our results supported the findings of Hussain et al. (2015) who presented that the seed inoculation of zinc-solubilizing isolates (*B. subtilis* and *B. megaterium*) enhanced zinc contents in wheat grains, which ultimately resulted in improved growth in both pot and field trials. Canola oil has the lowest saturated fat content of any vegetable oil; hence, it is preferred by diet-conscious consumers. Erucic acid and glucosinolates are harmful to both human and animal health, and they impart a bitter taste. The safe limits for these compounds are 30 μmol g^−1^ of glucosinolates and < 2% erucic acid in oil in oil-free diets (Moghadam et al., 2011). Our findings are consistent with those of Asghar et al. (2004), who similarly noticed a significant increase in oil content of *Brassica napus* L. by seed inoculation. However, in our study, the strains CLS2 and CLS9 demonstrated encouraging outcomes in terms of erucic acid and glucosinolate accumulation. There were low values of erucic acid in seeds inoculated with these strains. The above-mentioned results displayed the supportive role of inoculated strains in increasing oil and protein contents. Moreover, inoculation assisted the plants to maintain nutrition balance by reducing erucic acid and glucosinolate accumulations. One of the most limiting causes for low agricultural output is soil-borne pathogens. One of these mechanisms is the synthesis of siderophores, and these microbial compounds can lock iron away from pathogens (Kramer et al., 2020; Dar et al., 2021). As a result, cultivating region-specific microbial strains for the formation of most effective bioinoculum is required to maximize crop output and nutrient content of particular crop (Afzal et al., 2019). The use of harmless, ecologically safe, and beneficial PGPR as bioformulations has been suggested to be very beneficial in increasing agricultural productivity in a sustainable manner (Verma et al., 2019). It can also help us to cope with the problem of malnutrition by increasing Zn concentration in canola grains. These rhizobacteria could be used as biofertilizers to substitute agrochemicals in order to increase canola production, and this is in conformity with the findings of Zainab et al. (2021). Currently, limited reports are available on microorganisms that can transform insoluble forms of zinc into accessible forms. As a result, efforts were conducted to identify and evaluate the Zn-solubilizing bacteria from the canola rhizosphere for the purposes of growth stimulation and zinc biofortification.

## 5 Conclusion

Biofortification by inoculation of PGPR is one of the recently established alternatives for addressing agricultural issues brought on by a growing population, minimizing the use of chemical fertilizers, and contributing to development of environmentally friendly agriculture on a global level. The objective of this research was to isolate the zinc-solubilizing bacterial isolates from canola fields. The molecular identification of these isolates offers an advantage in obtaining a larger number of microorganisms from natural environments. As a result, these multi-trait isolates may be desirable inoculants for increasing canola yield and nutrient quality, hence reducing malnutrition. The isolated Zn-solubilizing bacteria improve nutrient utilization efficiency through a variety of mechanisms, including increased surface area accessible by plant roots and the synthesis of siderophores and exopolysaccharides. The majority of these bacteria could fix nitrogen, synthesize siderophores, IAA and solubilize zinc and phosphate. This research promotes the development of biotechnological techniques, such as inoculation with Zn-solubilizing bacteria that improves canola growth and yield. In the current study, selected rhizobacterial strains, *S. succinus* (CLS1), *P. aryabhattai* (CLS2), *B. subtilis* (CLS3), and *P. megaterium* (CLS9), have been demonstrated to be extremely effective zinc-solubilizing strains for seed inoculation, along with improvements in growth and yield attributes of canola in controlled as well as field conditions. This is the first study that reveals *S. succinus* as a Zn-solubilizing associated bacteria of canola plant. Most of the strains used in this study are not yet reported for zinc-solubilizing ability and their effect on canola growth and yield. These inoculants would be useful in improving oil quality and lowering the application of synthetic fertilizers in agriculture. The efficient rhizobacterial isolates identified in this study could be exploited to alleviate zinc deficiency, leading to a potential key for a sustainable canola crop production strategy.

## Data Availability

The datasets presented in this study can be found in online repositories. The names of the repository/repositories and accession number(s) can be found in the article/Supplementary material.

## Footnotes

^1^ http://macrogen.com/eng/
