# Supplementary figures and images for: Significance of zinc-solubilizing plant growth-promoting rhizobacterial strains in nutrient acquisition, enhancement of growth, yield, and oil content of canola (Brassica napus L.)
Source: Front Microbiol. 2024 Sep 27;15:1446064. doi: 10.3389/fmicb.2024.1446064 (PMC11466859; doi:10.3389/fmicb.2024.1446064)

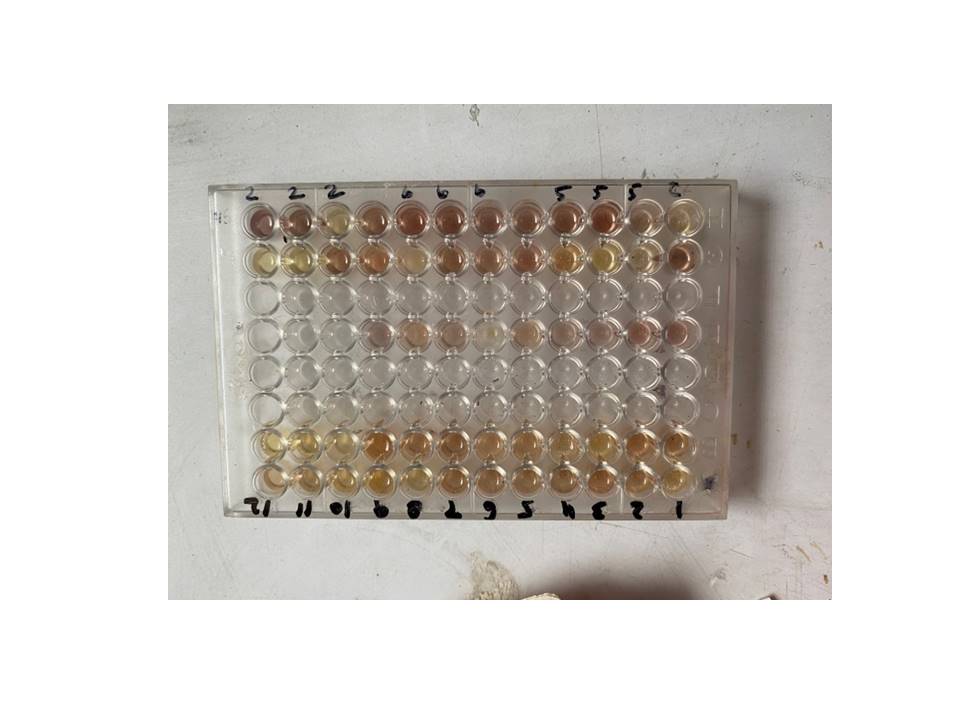

Supplement: Supplementary file 1 [file Image_1.JPEG]

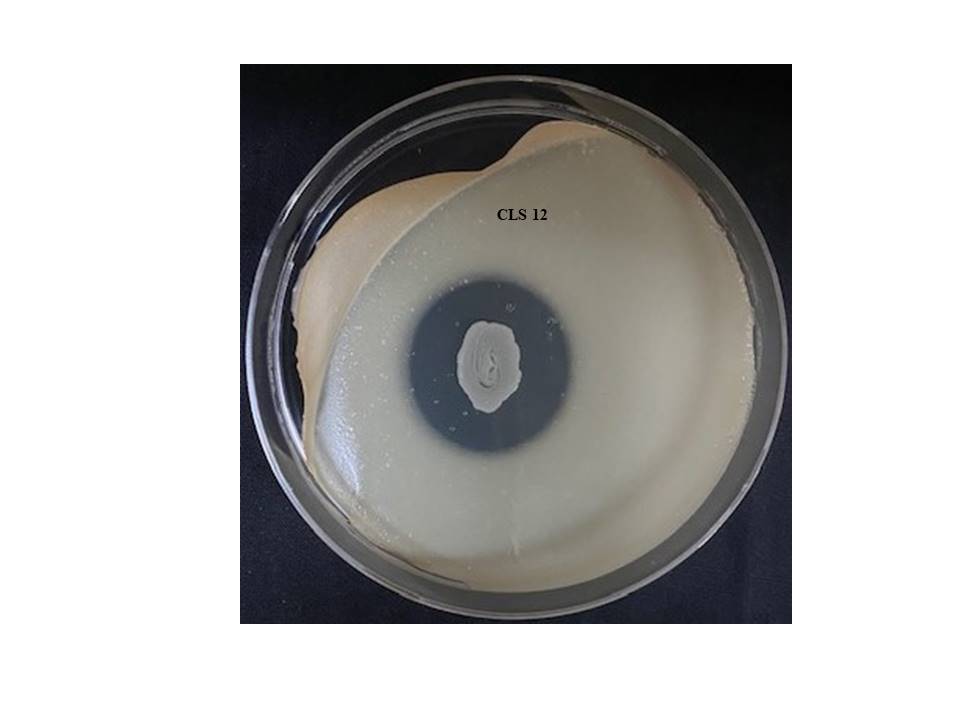

Supplement: Supplementary file 2 [file Image_2.JPEG]
