# Supplementary material for: Significance of zinc-solubilizing plant growth-promoting rhizobacterial strains in nutrient acquisition, enhancement of growth, yield, and oil content of canola (Brassica napus L.)
Source: Front Microbiol. 2024 Sep 27;15:1446064. doi: 10.3389/fmicb.2024.1446064 (PMC11466859; doi:10.3389/fmicb.2024.1446064)
Supplement: Supplementary file 3 [file Table_7.DOCX]

Figure P solubilization: Halo zones produced by phosphate solubilizing strains in Pikovskaya agar medium after 5 days of incubation. The largest halo zones were produced by CLS12.

Figure IAA production: IAA qualitative assay of rhizobacterial isolates CLS1-CLS16 in triplicate
